# Fast convergence of trust-regions for non-isolated minima via analysis of CG on indefinite matrices

**DOI:** 10.1007/s10107-024-02140-w

**Published:** 2024-10-18

**Authors:** Quentin Rebjock, Nicolas Boumal

**Affiliations:** Ecole Polytechnique Fédérale de Lausanne (EPFL), Insitute of Mathematics, Lausanne, Switzerland

**Keywords:** 90C26, 90C30, 65F22

## Abstract

Trust-region methods (TR) can converge quadratically to minima where the Hessian is positive definite. However, if the minima are not isolated, then the Hessian there cannot be positive definite. The weaker Polyak–Łojasiewicz (PŁ) condition is compatible with non-isolated minima, and it is enough for many algorithms to preserve good local behavior. Yet, TR with an *exact* subproblem solver lacks even basic features such as a capture theorem under PŁ. In practice, a popular *inexact* subproblem solver is the truncated conjugate gradient method (tCG). Empirically, TR-tCG exhibits superlinear convergence under PŁ. We confirm this theoretically. The main mathematical obstacle is that, under PŁ, at points arbitrarily close to minima, the Hessian has vanishingly small, possibly negative eigenvalues. Thus, tCG is applied to ill-conditioned, indefinite systems. Yet, the core theory underlying tCG is that of CG, which assumes a positive definite operator. Accordingly, we develop new tools to analyze the dynamics of CG in the presence of small eigenvalues of any sign, for the regime of interest to TR-tCG.

## Introduction

We consider unconstrained optimization problems of the form

$$\begin{aligned} \min _{x \in \mathcal {M}} f(x), \end{aligned}$$

where $\mathcal {M}$ is a Riemannian manifold^1^ and $f:\mathcal {M}\rightarrow {\mathbb R}$ is twice continuously differentiable (${\textrm{C}^{2}}$). The tangent spaces $\textrm{T}_x \mathcal {M}$ are equipped with inner products $\langle \cdot , \cdot \rangle _x$ and associated norms $\Vert \cdot \Vert _x$ (and we omit the subscript *x* for brevity).

Near a local minimum $\bar{x}$, classical results guarantee favorable local convergence properties for standard algorithms when the Hessian $\nabla ^2f(\bar{x})$ is positive definite. For example, gradient descent enjoys linear rates, while regularized variants of Newton’s method such as adaptive regularization with cubics (ARC) admit superlinear rates. Those algorithms preserve their fast convergence rates even if we relax the assumption at $\bar{x}$ to assume instead the weaker Polyak–Łojasiewicz (PŁ) condition around $\bar{x}$ (this is all well known: see Sect.  1.4 for a literature review).

### Definition 1.1

Let $\bar{x}$ be a local minimum of $f$. We say $f$ satisfies the *Polyak–Łojasiewicz* condition with constant $\mu > 0$ (also denoted $\mu$-*PŁ*) around $\bar{x}$ if 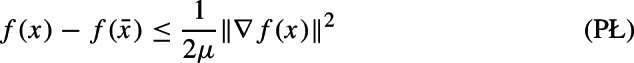 for all *x* in some neighborhood of $\bar{x}$, where $\nabla f$ is the gradient of $f$.

The situation is different for trust-region methods (TR). They are also known to enjoy superlinear convergence near local minima with a positive definite Hessian. However, in the literature there exist no fast (local) rates of convergence for TR algorithms assuming only the PŁ condition.

This is problematic if $f$ has non-isolated local minima, which is inevitable in overparameterized problems or problems with continuous symmetries. Indeed, the Hessian cannot be positive definite at non-isolated local minima, whereas PŁ can (and often does) hold there—see [33] and [37, Sect. 4.2], or [28] in the context of machine learning.

This gap in the literature is all the more surprising considering that *(i)* empirically, practical implementations of TR behave just fine near non-isolated minima with the PŁ condition, and *(ii)* the theoretical guarantees for TR usually parallel those of ARC, which is known to converge quadratically under PŁ [11, 37, 43, 49].

Both TR and ARC involve a subproblem at each iteration. For ARC, fast convergence rates hold under PŁ for various subproblem solvers, both exact and inexact. Surprisingly, for TR, solving the subproblems *exactly* can break basic capture properties under PŁ: we describe this in Sect. 1.2.

Practical implementations of TR seldom rely on an exact subproblem solver though. Instead, a popular alternative is the truncated conjugate gradient (tCG) method (see Algorithm 1). This is an *inexact* subproblem solver based on the conjugate gradient (CG) algorithm. The core of tCG is the same as CG but it terminates early if it detects negative curvature, or it exceeds the trust-region radius, or it produces a small enough residual.

Experimentally, we observe that TR with tCG enjoys favorable convergence properties around minima where PŁ holds (Appendix A provides a simple numerical example). The fact that this good behavior relies on a specific subproblem solver (which was not the case for ARC) may partly explain the literature gap—and highlights yet another remarkable property of Krylov methods.

In this paper, we explain that behavior theoretically. Specifically, we secure superlinear convergence (but not quadratic, see Remark 4.13) for TR with tCG under PŁ.

In our main theorem below, A1, A2 and A3 are three weak assumptions that typically hold, and that we describe in Sect. 4. For example, in the Euclidean case, they hold if the algorithm has access to the true Hessian and the latter is locally Lipschitz continuous. The proof is stated at the end of Sect. 4.

### Theorem 1.2

Suppose A1, A2, A3 and (PŁ) hold around a local minimum $\bar{x}$. We run TR with the tCG subproblem solver (Algorithm 1) with parameters $\kappa > 0$ and . Given any neighborhood $\mathcal {U}$ of $\bar{x}$, there exists a neighborhood $\mathcal {V}$ of $\bar{x}$ such that if an iterate enters $\mathcal {V}$ then the iterates converge superlinearly with order $1 + \theta$ to some local minimum of $f$ that is in $\mathcal {U}$.

To establish this theorem, we show several intermediate results of independent interest regarding Krylov methods. Indeed, a major obstacle to analyzing the behavior of tCG without assuming local strong convexity is that the Hessian can have small negative eigenvalues arbitrarily close to local minima, whereas the usual analyses of CG break if the Hessian is not positive definite. Accordingly, we propose a new analysis of CG that can handle ill-conditioned, indefinite matrices in Sects. 2 and 3.

**Table 1:** Simplifications in the case where $\mathcal {M}$ is a Euclidean space

	$\textrm{T}_x \mathcal {M}$	$\langle u, v\rangle$	$\Vert u\Vert$	$\textrm{R}_x(s)$	$\textrm{Exp}_x(s)$	$\textrm{Log}_x(y)$	${{\,\textrm{dist}\,}}(x, y)$
$\mathcal {M}= {\mathbb R}^n$	${\mathbb R}^n$	$u^\top v$	$\sqrt{u^\top u}$	$x + s$	$x + s$	$y - x$	$\Vert x - y\Vert$

### Sufficient conditions for superlinear local convergence

TR methods generate a sequence of iterates $x_k \in \mathcal {M}$ together with a sequence of trust-region radii $\Delta _k > 0$. At iteration *k*, a subproblem solver chooses a step $s_k$ in the tangent space $\textrm{T}_{x_k}\mathcal {M}$. Then, $x_{k+1}$ is set to be either $x_{k+1} = \textrm{R}_{x_k}(s_k)$ (accepted step) or $x_{k+1} = x_k$ (rejected step), where $\textrm{R}$ is a *retraction* on $\mathcal {M}$. (If $\mathcal {M}= {\mathbb R}^n$, then $\textrm{T}_{x}\mathcal {M}= {\mathbb R}^n$ and typically $\textrm{R}_{x}(s) = x+s$.) The step $s_k$ is chosen to approximately minimize a quadratic model $m_k :\textrm{T}_{x_k} \mathcal {M}\rightarrow {\mathbb R}$ under the constraint $\Vert s_k\Vert \le \Delta _k$, where

$$\begin{aligned} m_k(s) = f(x_k) + \langle s, \nabla f(x_k)\rangle + \frac{1}{2}\langle s, H_k[s]\rangle . \end{aligned}$$

Above, $H_k$ is a symmetric linear map (often set to be $\nabla ^2f(x_k)$) so that $m_k(s) \approx f(\textrm{R}_{x_k}(s))$ and $m_k(0) = f(x_k)$—Sect. 4 provides the details.

In Propositions 4.11 and 4.12, we identify three sufficient conditions on the subproblem solver’s choice of $s_k$ in order to secure superlinear local convergence of $\{x_k\}$ to a single point, assuming PŁ. Such conditions transpire in other proofs of superlinear convergence, see for example [2, Sect. 4.2] for TR and [49, Sect. 4] for regularized Newton.

The first condition is that $m_k(s_k)$ (which is approximately $f(x_{k+1})$ for accepted steps) is smaller than $m_k(0) = f(x_k)$, by some amount known as the *Cauchy decrease*.

#### C0

There exists a constant $c_0> 0$ such that, for all *k*, the step $s_k$ satisfies

$$\begin{aligned} m_k(0) - m_k(s_k) \ge c_0\Vert \nabla f(x_k)\Vert \min \!\bigg (\Delta _k, \frac{\Vert \nabla f(x_k)\Vert ^3}{\big |\langle \nabla f(x_k), H_k[\nabla f(x_k)]\rangle \big |}\bigg ). \end{aligned}$$

Essentially all reasonable subproblem solvers satisfy C0. The next two conditions control the behavior near local minima. Specifically, near a local minimum $\bar{x}$, steps should be small and be good approximate critical points of the model.

#### C1

There exist a constant $c_1\ge 0$ and a neighborhood $\mathcal {U}$ of $\bar{x}$ such that if an iterate $x_k$ is in $\mathcal {U}$ then the step $s_k$ satisfies

$$\begin{aligned} \Vert s_k\Vert \le c_1\Vert \nabla f(x_k)\Vert . \end{aligned}$$

#### C2

If the iterates converge to a local minimum, there exist constants $c_2\ge 0$ and $\theta > 0$ such that

$$\begin{aligned} \Vert \nabla m_k(s_k)\Vert \le c_2\Vert \nabla f(x_k)\Vert ^{1 + \theta } \end{aligned}$$

for all *k* large enough.

An *exact* subproblem solver certainly satisfies C0 [3, (7.14)]. If the iterates $x_k$ converge to a local minimum where the Hessian is positive definite, then an exact subproblem solver with $H_k = \nabla ^2f(x_k)$ also satisfies C1 and C2. This is because eventually the method produces Newton steps $s_k = -\nabla ^2f(x_k)^{-1}[\nabla f(x_k)]$ with $\Vert s_k\Vert \le \Vert \nabla ^2f(x_k)^{-1}\Vert \Vert \nabla f(x_k)\Vert < \Delta _k$, hence $\nabla m_k(s_k) = 0$. (See for example [3, (7.9) with $\mu = 0$].) Things are markedly different without the positive definite Hessian assumption, as we now discuss.

### Why an exact subproblem solver can fail yet tCG succeeds

In general, if we assume only the PŁ condition, exact subproblem solvers may fail C1. To understand why, it is useful to note that, for ${\textrm{C}^{2}}$ functions, the local PŁ condition is *equivalent* to the Morse–Bott ($\text {MB}$) property, as we define now—see [43, Sect. 2]. Let

1 $$\begin{aligned} \mathcal {S}&= \{x \in \mathcal {M}: x {\text { is a local minimum of }}\, f\} \end{aligned}$$

be the set that contains all the local minima of $f$. If $\mathcal {S}$ is an (embedded) submanifold of $\mathcal {M}$, then at each $\bar{x}\in \mathcal {S}$ it has a *tangent space* $\textrm{T}_{\bar{x}}\mathcal {S}$ whose orthogonal complement is the *normal space* $\textrm{N}_{\bar{x}}\mathcal {S}$.

#### Definition 1.3

We say $f$ satisfies the *Morse–Bott* property at a local minimum $\bar{x}\in \mathcal {S}$ if 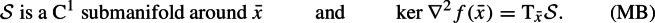 If also $\langle v, \nabla ^2f(\bar{x})[v]\rangle \ge \mu \Vert v\Vert ^2$ for some $\mu > 0$ and all $v \in \textrm{N}_{\bar{x}}\mathcal {S}$ then we say $f$ satisfies $\mu$-*MB* at $\bar{x}$.

**Fig. 1:**
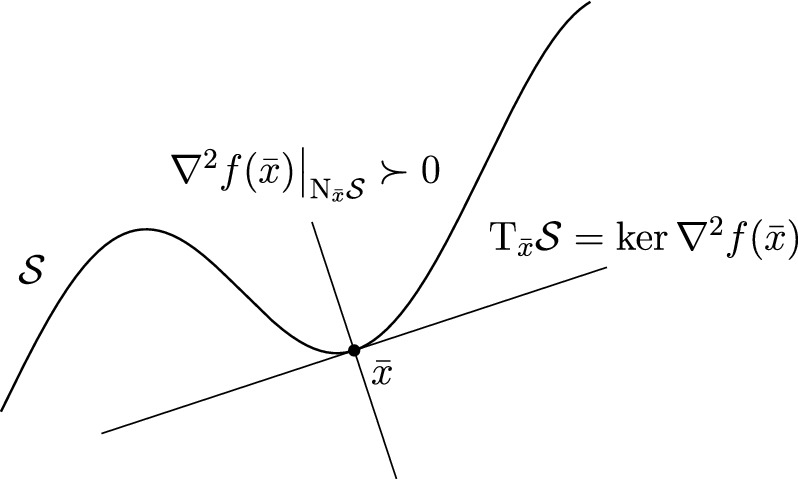
Illustration of the Morse–Bott property. The set of local minima $\mathcal {S}$ is smooth around the point $\bar{x}$. Here it has dimension 1 in the 2-dimensional search space $\mathcal {M}= {\mathbb R}^2$

Figure 1 illustrates the $\text {MB}$ property. Notice that the dimension of $\mathcal {S}$ as a manifold coincides with the dimension of the kernel of $\nabla ^2f(\bar{x})$. We now suppose that $f$ satisfies $\mu$-PŁ around a local minimum $\bar{x}$ and exploit the fact that this implies $\mu$-$\text {MB}$ at $\bar{x}$ to describe the behavior of both the exact subproblem solver and that of tCG around $\bar{x}$.

**The Hessian typically has small negative eigenvalues arbitrarily close to** $\bar{x}$. To see this, consider a small ball *B* around $\bar{x}$ and assume for contradiction that $\nabla ^2f$ is positive semidefinite at all points in *B*. If so, then $f$ restricted to *B* is (geodesically) convex, hence its minimizers form a convex set. Yet, the minimizers of $f$ restricted to *B* coincide with $\mathcal {S}\cap B$, and there is no reason a priori that this ought to be convex. Thus, save for that unusual case, there exist points *x* arbitrarily close to $\bar{x}$ where $\nabla ^2f(x)$ has a (small) negative eigenvalue. (Appendix A gives an explicit example.)

**Those eigenvalues defeat the exact subproblem solver**. Suppose that an iterate $x_k$ is at such a point where $\nabla ^2f(x_k)$ has a negative eigenvalue, and that $H_k = \nabla ^2f(x_k)$. This implies that the exact solution $s_k$ to the TR subproblem lies on the boundary of the trust region: $\Vert s_k\Vert = \Delta _k$ [38, Thm. 4.1]. This precludes capture results because the next iterate can be far ($\Delta _k$ may be large) even when $x_k$ is arbitrarily close to $\bar{x}$ (see [43, Sect. 4.2]). In particular, $\Vert s_k\Vert = \Delta _k$ is in general incompatible with condition C1. This issue occurs because the exact subproblem solver is highly sensitive to negative eigenvalues, even of small magnitude.

**Fig. 2:**
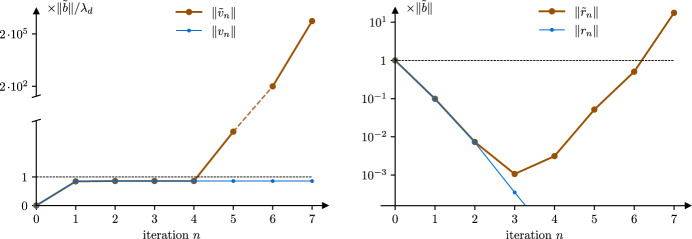
Norms of the iterates $\tilde{v}_n$ and residuals $\tilde{r}_n$ of CG on a problem $(\tilde{A}, \tilde{b})$. Here $\tilde{A}$ is diagonal with size ${\tilde{d}}= 11$. For illustration, there are $d= 10$ eigenvalues close to 1 and 1 eigenvalue equal to 0. The norm of the first $d$ entries of the weight vector $\tilde{b}$ is normalized to 1 and the entry associated to the zero eigenvalue is $10^{-3}$. Notice how the norm of the iterate $\tilde{v}_n$ explodes only *after* the residual $\tilde{r}_n$ became small: this is why tCG can stop *before* explosion, with a good solution. For reference, we also plot the same quantities for the well-conditioned problem $(A, b)$ of size 10, where the zero eigenvalue was removed

**But tCG automatically filters them out.** At $\bar{x}$, the Hessian has a kernel whose dimension is the same as that of $\mathcal {S}$ because ($\text {MB}$) holds. All the other eigenvalues are strictly positive. Given a point *x* close to $\bar{x}$, these positive eigenvalues remain large compared to the others, which are concentrated around zero. This defines a clear separation of the tangent space $\textrm{T}_x \mathcal {M}$ in two orthogonal complements: the primary space (large eigenvalues) and the secondary space (eigenvalues close to zero). Around $\bar{x}$, the gradient of $f$ is almost orthogonal to the secondary space (see Lemma 4.1). It follows that the Krylov space generated by $\nabla ^2f(x)$ and $\nabla f(x)$ essentially ignores the secondary components up to a certain number of iterations of CG. Beyond this critical number of iterations, the iterates of CG could explode because the algorithm detects the small eigenvalues of $\nabla ^2f(x)$. However, one of the key fact we secure in this paper is that the natural stopping criteria of tCG trigger *before* this explosion can happen (see Figure 2). We analyze these dynamics in Sect. 4.1, and deduce that tCG satisfies C1 and C2.

### Contributions

In the first part of the paper (Sects. 2 and 3), we design tools to study the dynamics of CG when the input matrix has eigenvalues of small magnitude that may be negative.

- From Sect. 1.2, we find that we need to understand the behavior of CG on systems $(\tilde{A}, \tilde{b})$ where $\tilde{A}$ may have some small (possibly negative) eigenvalues, and the corresponding components of $\tilde{b}$ are small. Existing theory does not handle that, but empirically we see that the initial iterates of CG on $(\tilde{A}, \tilde{b})$ are closely related to those of CG on $(A, b)$, where $A$ is the well-conditioned, positive definite part of $\tilde{A}$, and $b$ is the corresponding part of $\tilde{b}$. We make this precise in Theorem 3.9. See also Figure 2.
- In order to do this, we first relate the Lanczos polynomials associated to these two problems (Lemmas 3.2 and 3.3). We deduce sufficient conditions for an iteration of CG on $(\tilde{A}, \tilde{b})$ to be well defined (Lemma 3.4 and Corollary 3.5), in which case we relate the CG polynomials associated to the two problems (Theorem 3.6).
- That is only made possible by first introducing a special (and possibly new) family of polynomials in Sect. 2.2. We work out their properties with respect to the Lanczos polynomials.
- In Sect. 3.2 we particularize to a regime where the above results are actionable. In particular, we exhibit an iteration of CG with bounds for the iterate and residual norms (Lemma 3.14).

The second part (Sect. 4) of the paper applies the aforementioned results to TR-tCG.

- Our main result is Theorem 1.2. Given a local minimum where PŁ holds, it ensures capture of the iterates and superlinear convergence for TR with the tCG subproblem solver.
- Lemma 4.2 is the keystone to establish Theorem 1.2. The proof relies on Sect. 3. Roughly, it states that if *x* is a point near a local minimum where PŁ holds, the iterates of CG on $(\nabla ^2f(x), -\nabla f(x))$ are almost oblivious to the small eigenvalues of the Hessian, for a while.
- From Lemma 4.2 we deduce that TR with tCG satisfies conditions C1 and C2 around minima where PŁ holds (Propositions 4.5 and 4.7). This is because the early truncation rules of tCG trigger before the small eigenvalues can cause harm.
- Finally, in Sect. 4.2 we show that any subproblem solver that satisfies C0, C1 and C2 (tCG in particular) enables fast local convergence (Propositions 4.11 and 4.12).

As an aside, we deduce implications for optimization on quotient manifolds in Sect. 5.

### Related work

**Krylov subspace methods.** The history of Krylov methods is broad. [26] introduced his famous algorithm to find extreme eigenpairs of a matrix. Soon after, [24] described the CG algorithm to solve systems of linear equations. These two algorithms are tightly related. Standard references include [21] and [41]. It is classical to analyze Krylov subspace methods with polynomials [27, Ch. 2, 3]. This is because both the Lanczos and CG algorithms are linked to the theory of orthogonal polynomials and Gauss quadrature [18, Ch. 4].

Analyses of CG normally assume a positive definite matrix, whereas we need to handle (small) negative eigenvalues as well. There is a rich literature on CG with inexact arithmetic [20, 22, 39]. See [34] and [35, p. 475–476] for more pointers. Potentially, that line of work could have provided a foundation to build on. However, to the best of our knowledge, those analyses tend to assume sufficient positive definiteness to withstand rounding errors, and hence indefinite matrices are not (even indirectly) covered by their conclusions.

**Krylov and trust-region methods.** It is possible to solve the trust-region subproblem (TRS) exactly. Several algorithms have been proposed for this; for example, see [4, 36], and [10].

Solving the subproblem is only a means to an end (minimizing the cost function $f$). For this reason, many practical implementations of TR solve the subproblem only *approximately*. To do this, the Lanczos and CG algorithms (and variants) have been extensively used as subroutines. The truncated CG (tCG) algorithm is one particularly popular approach introduced by [46] and [45]. At the same time, [14, 15] proved that inexact Newton methods preserve fast convergence to non-degenerate critical points even with truncated steps. See also “notes and references” in [13, Sect. 7.5.1] for more historic notes. [19] proposed an algorithm to continue the tCG process after the algorithm reaches the boundary of the trust region. [48] proved that the model decrease resulting from the tCG step is at least half that of the exact solution when the Hessian is positive definite.

The minimal residual method (MINRES, introduced by [40]) is closely related to CG. [17] empirically compared CG and MINRES. They suggested that MINRES may be preferable when the algorithm is meant to stop early (as is the case for the TRS), and they proposed partial theoretical explanations. Later, [29, 30] scrutinized the preeminence of CG to solve subproblems in Newton-type methods and also argued that MINRES is a reasonable choice. They proved that MINRES enjoys many favorable properties and proposed a Newton-type algorithm based on it. Analyzing the local behavior of MINRES under the PŁ condition could be an interesting direction to extend our work.

**PŁ and local convergence.** In seminal articles, [31, 32] showed functions in a large class satisfy special inequalities, and he used them to study continuous dynamical systems. Concurrently, [42] proved that a global version of those inequalities is sufficient for gradient descent to converge linearly. Since then, the condition is often called PŁ (also “gradient dominance”). Variations have been extensively invoked in optimization literature, notably [5, 6, 8, 25]. We focus here on the interactions between PŁ and second-order algorithms.

[37] popularized the regularized Newton algorithm of [23] and proved local superlinear convergence of order 4/3 assuming PŁ. Later, [11, 12] proposed an adaptive variant (ARC) and proved quadratic convergence to points where the Hessian is positive definite. [50] characterized the local convergence rate of regularized Newton depending on the Łojasiewicz exponent of the problem. [49] proved that regularized Newton with exact subproblem solver converges quadratically to minimizers where the *error bound* condition holds. This condition is in fact equivalent to PŁ; [43] leveraged this to obtain quadratic convergence for ARC, with both exact and inexact subproblem solvers.

In contrast, for trust-region methods there exist superlinear convergence results in two cases: *(i)* for general $f$ with positive definite Hessian (see for example [38, Sect. 4.4] and [2]), and *(ii)* assuming PŁ but specialized to nonlinear least squares $x \mapsto \Vert F(x)\Vert ^2$, around a global minimum $\bar{x}$ satisfying $F(\bar{x}) = 0$ [16]. In that work, the subproblems are modified in a Levenberg–Marquardt way, which ensures semidefinite Hessian approximations.

## CG: reminders and a possibly new family of polynomials

This section collects properties of CG we need in Sect. 3. The first part recalls classical theory. The second part introduces key polynomials. In this whole section, we let $A$ be a $d\times d$ symmetric matrix and $b\in {\mathbb R}^d$. Since CG is equivariant with respect to orthogonal transformations, we assume without loss of generality that $A$ is diagonal, that is,

$$\begin{aligned} A= {{\,\textrm{diag}\,}}(\lambda _1, \dots , \lambda _d)  &   \qquad {\text {where}} \qquad  &   \lambda _1 \ge \dots \ge \lambda _d. \end{aligned}$$

However, we do *not* assume that the input matrix $A$ is positive definite nor that it is well conditioned. We consider exact arithmetic.

Many of the results below rely on polynomials (summarized in Table 2). The set $\mathcal {P}_{\le n}$ contains all univariate polynomials with real coefficients and of degree at most *n*. Given a polynomial $p$, we let $\vartheta _{n}(p)$ denote its coefficient of degree *n*. Furthermore,  is the restriction of a linear operator $A$ to a subspace $\mathcal {K}$.

### Background: CG through the lens of optimal polynomials

**Algorithm 1:**
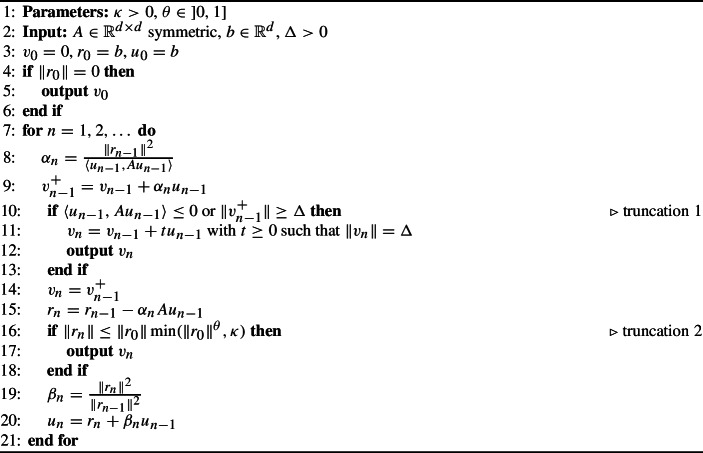
Truncated conjugate gradient (tCG)

Algorithm 1 describes tCG. CG is the same but without the two truncation parts in lines 10–13 and 16–18. We always consider that the starting vector is $v_0 = 0$. This section provides some classical background about CG. Given an integer $n \in \{1, \dots , d\}$, we define the *n*th Krylov matrix and the associated subspace as

$$\begin{aligned} K_n = \begin{bmatrix} b&Ab&\cdots&A^{n - 1} b\end{bmatrix}  &   {\text {and}}  &   \mathcal {K}_n = {{\,\textrm{span}\,}}K_n. \end{aligned}$$

#### Definition 2.1

The *grade* $\ell = \ell (A, b)$ is the largest integer *n* such that $\mathcal {K}_n$ has dimension *n*.

#### Definition 2.2

The *n*th iteration of CG is *well defined* if $n \le \ell$ and $A|_{\mathcal {K}_n} \succ 0$.

Iteration *n* is well defined exactly if the inner products in line 8 are (strictly) positive up to the *n*th iteration [47, Thm. 38.1]. We consider only those iterations.

**Lanczos polynomials.** CG is an iterative procedure to solve $Ax = b$. The Lanczos algorithm is an iterative procedure to compute extreme eigenpairs. Their iterations are related to a sequence of monic orthogonal polynomials that we describe now. Define the bilinear form

2 $$\begin{aligned} \langle p, q\rangle = \sum _{i = 1}^db_i^2 p(\lambda _i)q(\lambda _i) \end{aligned}$$

on $\mathcal {P}_{\le \ell }$ (the linear space of polynomials of degree up to $\ell$). It is a semi-inner product which is positive definite on $\mathcal {P}_{\le \ell - 1}$. The form $\langle \cdot , \cdot \rangle$ induces a semi-norm $\Vert \cdot \Vert$ on $\mathcal {P}_{\le \ell }$. With $\vartheta _{n}(p)$ denoting the coefficient of degree *n* for the polynomial *p*, the *n*th *Lanczos polynomial* is the (unique) solution to the convex optimization problem

3 $$\begin{aligned} \pi _n = \mathop {\mathrm {arg\,min}}\limits _{\pi \in \mathcal {P}_{\le n}} \Vert \pi \Vert ^2 \qquad {\text {subject to}} \qquad \vartheta _{n}(\pi ) = 1. \end{aligned}$$

In particular, $\pi _n$ is monic of degree *n*. The kernel of $\langle \cdot , \cdot \rangle$ is ${{\,\textrm{span}\,}}\pi _\ell$. The following lemma states that the polynomials $\{\pi _i\}$ are well defined and it describes some of their properties.

#### Lemma 2.3

For all $n \in \{0, \dots , \ell \}$ there exists a unique solution $\pi _n$ to (3). The *n* roots of $\pi _n$ are real, distinct, and in the interval . The polynomials $\pi _0, \dots , \pi _\ell$ are orthogonal for (2).

#### Proof

This is classical: see for example [27, Lem. 3.2.2, Lem. 3.2.4]. $\square$

It is also known that the roots of $\pi _n$ and $\pi _{n + 1}$ interlace but we are not going to use this fact.

#### Remark 2.4

Given an eigenvalue $\lambda$ of $A$, let $P_\lambda$ be the orthogonal projector onto the eigenspace of $A$ associated to $\lambda$. Define the *weight* of $\lambda$ as $\Vert P_\lambda b\Vert$. The grade $\ell$ of $(A, b)$ is the number of distinct eigenvalues of $A$ with (strictly) positive weight. It is also the smallest integer *n* such that $\Vert \pi _n\Vert = 0$.

With the orthogonality of $\pi _0, \dots , \pi _\ell$ and a simple application of the Pythagorean theorem we obtain the following decomposition.

#### Lemma 2.5

Given $n \in \{0, \dots , \ell \}$ and a polynomial $p\in \mathcal {P}_{\le n}$, the following decomposition holds:

$$\begin{aligned} p= \vartheta _{n}(p) \pi _n + \sum _{i = 0}^{n - 1} \frac{\langle p, \pi _i\rangle }{\Vert \pi _i\Vert ^2}\pi _i. \end{aligned}$$

#### Proof

The polynomial $q = p - \vartheta _{n}(p)\pi _n$ has degree at most $n - 1$. Decompose *q* in the orthogonal basis $\{\pi _0, \dots , \pi _{n - 1}\}$ (Lemma 2.3) and leverage $\langle \pi _n, \pi _i\rangle = 0$ for all $i < n$ to obtain the identity. $\square$

The optimality conditions of the convex optimization problem (3) entirely characterize the polynomial $\pi _n$.

#### Lemma 2.6

For $n \in \{0, \dots , \ell \}$, the polynomial $\pi _n$ defined in (3) is uniquely characterized by

4 $$\begin{aligned} \langle p, \pi _n\rangle = \vartheta _{n}(p)\Vert \pi _n\Vert ^2 \end{aligned}$$

for all $p\in \mathcal {P}_{\le n}$. In particular, $\langle p, \pi _n\rangle = 0$ when $\deg p\le n - 1$.

#### Proof

Problem (3) is convex with a unique solution for the given *n*. It is hence characterized by the first-order optimality conditions: $\langle q, \pi _n\rangle = 0$ for all $q \in \mathcal {P}_{\le n - 1}$. Notice that a polynomial $p\in \mathcal {P}_{\le n}$ can be written $p= \vartheta _{n}(p) \pi _n + q$ where $q \in \mathcal {P}_{\le n - 1}$. Plugging this in the optimality condition gives $\langle p, \pi _n\rangle = \vartheta _{n}(p) \Vert \pi _n\Vert ^2$. $\square$

The roots of $\pi _n$ are called the *Ritz values*. The following classical lemma [47, Thm. 36.1] links them to the eigenvalues of $A$ restricted to the *n*th Krylov subspace.

#### Lemma 2.7

For all $n \le \ell$ the roots of $\pi _n$ coincide with the eigenvalues of .

**Connections to the CG algorithm.** The polynomials $\{\pi _i\}$ are related to the iterations of CG as we describe now; see [27, Sect. 5.6] for details. Given $n \le \ell$, the *n*th iteration of CG is well defined (Definition 2.2) if and only if  is positive definite. This is equivalent to $\pi _n$ having only positive roots by Lemma 2.7. In this case, we define the degree *n* polynomial

5 $$\begin{aligned} \varsigma _n = \frac{\pi _n}{\pi _n(0)}. \end{aligned}$$

This is a rescaled version of $\pi _n$ such that $\varsigma _n(0) = 1$. Moreover, let $\varphi _{n - 1}$ be the degree $n - 1$ polynomial that satisfies $\varsigma _n(x) = 1 - x \varphi _{n - 1}(x)$. We can understand the state of CG at iteration *n* from the polynomials $\varsigma _n$ and $\varphi _{n - 1}$. Indeed, the *n*th iterate and residual of CG are given by

$$\begin{aligned} v_n = \varphi _{n - 1}(A)b  &   \qquad {\text {and}} \qquad  &   r_n = \varsigma _n(A)b\end{aligned}$$

respectively. It also follows that the norm of the residual is $\Vert r_n\Vert = \Vert \varsigma _n\Vert$, where the norm on the right-hand side is the one induced by (2).

**CG is a minimization algorithm.** As long as the matrix $A$ is positive definite on the *n*th Krylov subspace $\mathcal {K}_n$, the iterates $v_n$ of CG minimize a quadratic form as follows [27, Sect. 2.5.3]:

6 $$\begin{aligned} v_n = \mathop {\mathrm {arg\,min}}\limits _{v \in \mathcal {K}_n} \frac{1}{2} v^\top Av - b^\top v  &   \qquad {\text {and}} \qquad  &   r_n = b- Av_n. \end{aligned}$$

Let the columns of $Q_n \in {\mathbb R}^{d\times n}$ be an orthonormal basis of ${\mathcal {K}}_n$. Then from (6) we can write $v_n = Q_n(Q_n^\top AQ_n )^{-1}Q_n^\top b$. The eigenvalues of $Q_n^\top AQ_n$ are the Ritz values, that is, the roots of $\pi _n$ owing to Lemma 2.7. It follows that the spectrum of the matrix $Q_n(Q_n^\top AQ_n )^{-1}Q_n^\top$ contains $d- n$ zeros and the other eigenvalues are the inverses of the roots of $\pi _n$.

When the input matrix $A$ is positive definite we can see the polynomials $\{\varsigma _i\}$ as the solutions of particular optimization problems. Remember that we do *not* assume that $A$ is positive definite in this section. However, we will apply this result to a positive definite submatrix in Sect. 3.2.

#### Lemma 2.8

Assume that $\lambda _1, \dots , \lambda _d> 0$. For all $n \in \{0, \dots , \ell \}$ the polynomial $\varsigma _n$, as defined in (5), is the unique solution to the optimization problem

7 $$\begin{aligned} \min _{\varsigma \in \mathcal {P}_{\le n}} \; \sum _{i = 1}^d\frac{\varsigma (\lambda _i)^2}{\lambda _i} b_i^2 \qquad {\text {subject to}} \qquad \varsigma (0) = 1. \end{aligned}$$

#### Proof

Problem (7) is convex. The first-order optimality conditions state that a feasible polynomial $\varsigma$ is optimal if and only if $\sum _{i = 1}^d\lambda _i^{-1} p(\lambda _i) \varsigma (\lambda _i) b_i^2 = 0$ for all $p \in \mathcal {P}_{\le n}$ satisfying $p(0) = 0$. The latter requires that $p(x) = x q(x)$ for some $q \in \mathcal {P}_{\le n - 1}$, hence $\varsigma$ is optimal if and only if $\sum _{i = 1}^dq(\lambda _i) \varsigma (\lambda _i) b_i^2 = 0$ for all $q \in \mathcal {P}_{\le n - 1}$. We conclude with Lemma 2.6. $\square$

**Table 2:** Summary of polynomials used in Sects. 2 and 3

Polynomial	Definition	Normalization	Comments	Relation to CG
$$\pi _n$$				
$\tilde{\pi }_n$	Minimizers of (15)	Monic	Lemmas 2.3, 2.7 and 3.3	
$\varsigma _n$	$\varsigma _n = \pi _n/\pi _n(0)$	$\varsigma _n(0) = 1$	Minimizer of (7)	$r_n = \varsigma _n(A) b$
$\tilde{\varsigma }_n$	$\tilde{\varsigma }_n = \tilde{\pi }_n/\tilde{\pi }_n(0)$	$\tilde{\varsigma }_n(0) = 1$	Theorem 3.6	$\tilde{r}_n = \tilde{\varsigma }_n(\tilde{A}) \tilde{b}$
$\varphi _{n - 1}$	$\varsigma _n(x) = 1 - x \varphi _{n - 1}(x)$		Lemma 3.7	$v_n = \varphi _{n - 1}(A) b$
$\tilde{\varphi }_{n - 1}$	$\tilde{\varsigma }_n(x) = 1 - x \tilde{\varphi }_{n - 1}(x)$	none		$\tilde{v}_n = \tilde{\varphi }_{n - 1}(\tilde{A}) \tilde{b}$
$\zeta _n$	Minimizer of (8)	$\zeta _n(\lambda ) = 1$	$\zeta _n^j = \zeta _n$ with $\lambda = \lambda _j$	
$\xi _n$	$\xi _n = \zeta _n/\zeta _n(0)$	$\xi _n(0) = 1$	Interlace with $\pi _{n + 1}$	

### A possibly new family of polynomials

In this section, we introduce special polynomials $\{\zeta _i\}$ that enjoy convenient properties with respect to the Lanczos polynomials $\{\pi _i\}$ defined in (3). We have not seen these polynomials studied elsewhere, though they are intimately related to the minimal residual method (MINRES).^2^ Their extremal and interlacing properties play a key role to understand CG on two related instances in Sect. 3. Remember that Table 2 summarizes all the polynomials that we manipulate.

Fix some $\lambda \in {\mathbb R}$ for this whole section. (Later, we invoke its results for various values of $\lambda$.) Then, for each $n < \ell$ let

8 $$\begin{aligned} \zeta _n = \mathop {\mathrm {arg\,min}}\limits _{\zeta \in \mathcal {P}_{\le n}} \Vert \zeta \Vert ^2 \qquad {\text {subject to}} \qquad \zeta (\lambda ) = 1. \end{aligned}$$

There is indeed a unique solution to (8) when $n < \ell$ because $\Vert \cdot \Vert ^2$ is strongly convex on $\mathcal {P}_{\le \ell - 1}$. (We do not need this, but when $n = \ell$ there is also a unique solution provided that $\lambda \notin \{\lambda _1, \dots , \lambda _d\}$.)

We show below that $\zeta _n$ is of degree *n* and that its roots are real and distinct. We first derive some basic properties. The optimality conditions of the convex optimization problem (8) entirely characterize the polynomial $\zeta _n$.

#### Lemma 2.9

For $n \in \{0, \dots , \ell - 1\}$, the polynomial $\zeta _n$ defined in (8) is uniquely characterized by

9 $$\begin{aligned} \langle p, \zeta _n\rangle = p(\lambda )\Vert \zeta _n\Vert ^2 \end{aligned}$$

for all $$p\in \mathcal {P}_{\le n}$$.

#### Proof

Problem (8) is strongly convex for the given *n*, hence its unique solution is characterized by the first-order optimality conditions. Explicitly, $\langle q, \zeta _n\rangle = 0$ for all $q \in \mathcal {P}_{\le n}$ such that $q(\lambda ) = 0$. Notice that a polynomial $p\in \mathcal {P}_{\le n}$ can be written $p= p(\lambda ) \zeta _n + q$ where $q \in \mathcal {P}_{\le n}$ satisfies $q(\lambda ) = 0$. Plugging this in the optimality condition gives $\langle p, \zeta _n\rangle = p(\lambda )\Vert \zeta _n\Vert ^2$. $\square$

#### Lemma 2.10

The identity

10 $$\begin{aligned} \frac{\zeta _n}{\Vert \zeta _n\Vert ^2} = \frac{\zeta _{n - 1}}{\Vert \zeta _{n - 1}\Vert ^2} + \pi _n(\lambda )\frac{\pi _n}{\Vert \pi _n\Vert ^2} \end{aligned}$$

holds for all $n \in \{1, \dots , \ell - 1\}$.

#### Proof

Decompose the polynomial $\zeta _n$ in the orthogonal basis $\{\pi _0, \dots , \pi _n\}$ (Lemma 2.3) to obtain

$$\begin{aligned} \zeta _n = \sum _{i = 0}^n \frac{\langle \zeta _n, \pi _i\rangle }{\Vert \pi _i\Vert ^2} \pi _i = \Vert \zeta _n\Vert ^2 \sum _{i = 0}^n \frac{\pi _i(\lambda )}{\Vert \pi _i\Vert ^2}\pi _i, \end{aligned}$$

where we used the optimality condition (9) for the second equality. Divide by $\Vert \zeta _n\Vert ^2$ and subtract two consecutive equalities to obtain the result. $\square$

The following is a variation of classical arguments at the basis of the Cristoffel–Darboux formula. This is an important result to control the roots of the polynomials $\{\zeta _i\}$.

#### Lemma 2.11

Let $n \in \{0, \dots , \ell - 1\}$. If $\lambda < \lambda _d$ then the roots of $\zeta _n$ and $\pi _{n + 1}$ interlace (strictly). In particular, $\zeta _n$ has degree *n*, and its roots are real, distinct, and in the interval .

#### Proof

It is sufficient to show that $\pi _{n + 1}'\zeta _n - \pi _{n + 1}\zeta _n'$ has constant (strict) sign to obtain that the roots interlace. Indeed, assume it is the case and consider two consecutive zeros $z_1 < z_2$ of $\pi _{n + 1}$. Rolle’s theorem implies that $\pi _{n + 1}'(z_1)$ and $\pi _{n + 1}'(z_2)$ have opposite signs (recall that the zeros of $\pi _{n + 1}$ are simple by Lemma 2.3). Since $\pi _{n + 1}'\zeta _n - \pi _{n + 1}\zeta _n'$ has constant sign, it follows that $\zeta _n(z_1)\zeta _n(z_2) < 0$. This implies that $\zeta _n$ has a root in the interval .

We now prove by induction that the polynomial $\pi _{n + 1}'\zeta _n - \pi _{n + 1}\zeta _n'$ indeed has constant sign equal to $(-1)^n$ for all $n \in \{0, \dots , \ell - 1\}$. The claim is clear when $n = 0$ because $\pi _1'\zeta _0 - \pi _1\zeta _0' = 1$. Let $n \in \{1, \dots , \ell - 1\}$ and suppose that the claim is true at order $n - 1$. Define the polynomial $p:x \mapsto (x - \lambda )\zeta _n(x)$ and decompose it in the orthogonal basis $\{\pi _0, \dots , \pi _{n + 1}\}$ with Lemma 2.5. For all $i < n$ observe that $\langle p, \pi _i\rangle = \langle \zeta _n, (x - \lambda )\pi _i\rangle = 0$ using the characterization of $\zeta _n$ in (9). We obtain

11 $$\begin{aligned} p= \alpha _n \pi _{n + 1} + \beta _n \pi _n \end{aligned}$$

where $\alpha _n = \vartheta _{n + 1}(p)$ and $\beta _n = \langle p, \pi _n\rangle /\Vert \pi _n\Vert ^2$. From (11) we deduce that

12 $$\begin{aligned} \begin{aligned} (x - \lambda )\zeta _n(x)\zeta _n(y) = \alpha _n\pi _{n + 1}(x)\zeta _n(y) + \beta _n\pi _n(x)\zeta _n(y)\\ (y - \lambda )\zeta _n(y)\zeta _n(x) = \alpha _n\pi _{n + 1}(y)\zeta _n(x) + \beta _n\pi _n(y)\zeta _n(x) \end{aligned} \end{aligned}$$

for all *x*, *y*. From the identity (10) and the definition of *p* we find that $\alpha _n = \pi _n(\lambda )\Vert \zeta _n\Vert ^2/\Vert \pi _n\Vert ^2$, which is a non-zero number (see Lemma 2.3). Subtracting the two identities in (12) and dividing by $\alpha _n(x - y)$ yields

$$\begin{aligned}&\frac{\pi _{n + 1}(x)\zeta _n(y) - \pi _{n + 1}(y)\zeta _n(x)}{x - y} \\&\quad = \frac{1}{\alpha _n}\bigg (\zeta _n(x)\zeta _n(y) - \beta _n\frac{\pi _n(x)\zeta _n(y) - \pi _n(y)\zeta _n(x)}{x - y}\bigg )\\&\quad = \frac{1}{\alpha _n}\bigg (\zeta _n(x)\zeta _n(y) - \beta _n\frac{\Vert \zeta _n\Vert ^2}{\Vert \zeta _{n - 1}\Vert ^2}\frac{\pi _n(x)\zeta _{n - 1}(y) - \pi _n(y)\zeta _{n - 1}(x)}{x - y}\bigg ), \end{aligned}$$

where we used (10) for the last equality. Taking the limit $y \rightarrow x$ in the previous expression gives

13 $$\begin{aligned} \pi _{n + 1}'\zeta _n - \pi _{n + 1}\zeta _n' = \frac{1}{\alpha _n}\bigg (\zeta _n^2 - \beta _n \frac{\Vert \zeta _n\Vert ^2}{\Vert \zeta _{n - 1}\Vert ^2}\big ( \pi _n'\zeta _{n - 1} - \pi _n\zeta _{n - 1}' \big )\bigg ). \end{aligned}$$

We now study the signs of $\alpha _n$ and $\beta _n$. Recall that Lemma 2.3 ensures that the degree *n* polynomial $\pi _n$ is monic with roots in the interval . We assumed $\lambda < \lambda _d$ so this implies ${{\,\textrm{sign}\,}}\pi _n(\lambda ) = (-1)^n$. From the expression $\alpha _n = \pi _n(\lambda )\Vert \zeta _n\Vert ^2/\Vert \pi _n\Vert ^2$ we deduce ${{\,\textrm{sign}\,}}\alpha _n = {{\,\textrm{sign}\,}}\pi _n(\lambda ) = (-1)^n$. Now consider $\beta _n = \langle p, \pi _n\rangle /\Vert \pi _n\Vert ^2$. We substitute $\pi _n / \Vert \pi _n\Vert ^2$ using (10) and notice that (9) implies $\langle (x - \lambda ) \zeta _n, \zeta _{n - 1}\rangle = \langle \zeta _n, (x - \lambda ) \zeta _{n - 1}\rangle = 0$. This yields

$$\begin{aligned} \beta _n = \frac{1}{\pi _n(\lambda )\Vert \zeta _n\Vert ^2}\langle p, \zeta _n\rangle = \frac{1}{\pi _n(\lambda )\Vert \zeta _n\Vert ^2}\sum _{i = 1}^db_i^2 (\lambda _i - \lambda ) \zeta _n(\lambda _i)^2, \end{aligned}$$

where the second equality comes from the definition of the inner product $\langle \cdot , \cdot \rangle$. At least one of the terms in the sum is (strictly) positive because $\zeta _n$ has degree at most $n < \ell$ (see Remark 2.4). It follows that ${{\,\textrm{sign}\,}}\beta _n = {{\,\textrm{sign}\,}}\pi _n(\lambda ) = (-1)^n$. We deduce from (13) that the sign of $\pi _{n + 1}'\zeta _n - \pi _{n + 1}\zeta _n'$ is constant and equal to $(-1)^n$. $\square$

In fact, the conclusion of Lemma 2.11 remains true for $\lambda > \lambda _1$ but we will not need this.

## Understanding CG with eigenvalues around zero

In this section we build tools to analyze CG when the input matrix has a small (possibly) indefinite part with eigenvalues close to zero. To do this, we relate the behavior of CG on two instances. Specifically, we let ${\tilde{d}}\ge d$ be two integers. Given $\lambda _{1\phantom {{\tilde{d}}}}, \dots , \lambda _{\tilde{d}}\in {\mathbb R}$ and $b_{1\phantom {{\tilde{d}}}}, \dots , b_{\tilde{d}}\in {\mathbb R}$, we define

$$\begin{aligned} A&= {{\,\textrm{diag}\,}}(\lambda _1, \dots , \lambda _d), \qquad \qquad \qquad \qquad \qquad \qquad \quad b= (b_1, \dots , b_d)^\top ,\\ \tilde{A}&= {{\,\textrm{diag}\,}}(\lambda _{1\phantom {{\tilde{d}}}}, \dots , \lambda _{d\phantom {{\tilde{d}}}}, \lambda _{d+ 1\phantom {{\tilde{d}}}}, \dots , \lambda _{\tilde{d}}), \qquad {\text {and}}\qquad \tilde{b}= (b_{1\phantom {{\tilde{d}}}}, \dots , b_{d\phantom {{\tilde{d}}}}, b_{d+ 1\phantom {{\tilde{d}}}}, \dots , b_{\tilde{d}})^\top . \end{aligned}$$

**Fig. 3:**
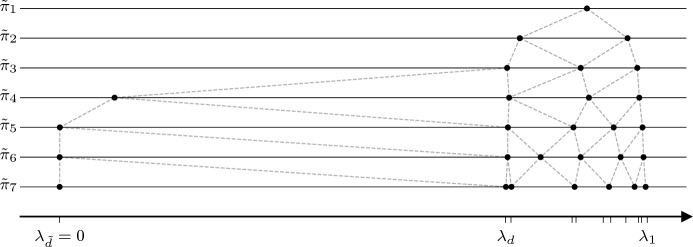
Lanczos polynomials $\tilde{\pi }_1, \ldots , \tilde{\pi }_7$ associated to the iterates 1 to 7 for the problem in Figure 2. The horizontal axis shows the eigenvalues $\lambda _1 \ge \dots \ge \lambda _d> \lambda _{\tilde{d}}= 0$ of $\tilde{A}$. For each iteration *n* we plot the roots of the Lanczos polynomial $\tilde{\pi }_n$. The lines linking the roots emphasize the interlacement. Most of the weight of $\tilde{b}$ lies in the interval . This is why the roots are located in this interval during the first iterations. After the fourth iteration the minimal root rapidly approaches zero. This causes the explosion of the iterates and residuals (see Figure 2), but tCG can stop earlier

As a result, $A$ is a submatrix of $\tilde{A}$ and $b$ is the corresponding subvector of $\tilde{b}$. We think of $(A, b)$ as a well-behaved reference problem (later we will assume $A\succ 0$) and we want to understand CG on $(\tilde{A}, \tilde{b})$, where $\tilde{A}$ may have an indefinite part. To do this, we relate the Lanczos polynomials of the two problems in Sect. 3.1. This relation relies on the special polynomials introduced in Sect. 2.2 and involves a specific vector $\sigma$ of size ${\tilde{d}}- d$. Since the Lanczos polynomials determine the iterates of CG (Sect. 2.1), we can deduce a relation between the iterates of the two problems. We find that they are close when the 1-norm of $\sigma$ is small. Finally, in Sect. 3.2 we consider a certain regime and secure that this norm is indeed small at some iteration. Figures 2 and 3 illustrate the empirical behavior of CG on two such instances.

### Tracking CG iterates on two related problems

In this section, we seek to understand the iterations of CG with inputs $(\tilde{A}, \tilde{b})$ as they relate to those with inputs $(A, b)$. We let $\ell$ denote the grade of $(A, b)$. Note that the grade of $(\tilde{A}, \tilde{b})$ is at least $\ell$.

**A link between the Lanczos polynomials.** We define two semi-inner products $\langle \cdot , \cdot \rangle$ and $\langle \cdot , \cdot \rangle _\sim$ on $\mathcal {P}_{\le \ell }$ as

14 $$\begin{aligned} \langle p, q\rangle = \sum _{i = 1}^db_i^2 p(\lambda _i)q(\lambda _i) \quad {\text {and}} \quad \langle p, q\rangle _\sim = \sum _{i = 1}^{\tilde{d}}b_i^2 p(\lambda _i)q(\lambda _i) = \langle p, q\rangle + \sum _{i = d+ 1}^{\tilde{d}}b_i^2 p(\lambda _i)q(\lambda _i).\nonumber \\ \end{aligned}$$

They induce the two semi-norms $\Vert \cdot \Vert$ and $\Vert \cdot \Vert _\sim$. We can then define the Lanczos polynomials of degree $n \le \ell$ associated to each problem as

15 $$\begin{aligned} \pi _n = \mathop {\mathrm {arg\,min}}\limits _{\pi \in \mathcal {P}_{\le n}} \Vert \pi \Vert ^2  &   {\text {and}}  &   \tilde{\pi }_n = \mathop {\mathrm {arg\,min}}\limits _{\pi \in \mathcal {P}_{\le n}} \Vert \pi \Vert _\sim ^2,  &   {\text {both subject to}}  &   \vartheta _{n}(\pi ) = 1. \end{aligned}$$

To understand the iterations of CG on $(\tilde{A}, \tilde{b})$, we set out to relate $\pi _n$ and $\tilde{\pi }_n$ using the polynomials $\{\zeta _i\}$ defined in Sect. 2.2. For this, we rely on ${\tilde{d}}- d$ instances of them.

#### Definition 3.1

For $d< j \le {\tilde{d}}$ and $n < \ell$, let $\zeta _n^j$ be the solution to (8) with data $(A, b)$ and $\lambda = \lambda _j$.

The conclusion of the following lemma holds without any assumption on the eigenvalues $\lambda _1, \dots , \lambda _{{\tilde{d}}}$. In particular, we do *not* assume that they are positive. For convenience, $B, C, D \in {\mathbb R}^{({\tilde{d}}- d) \times ({\tilde{d}}- d)}$ and $w, \sigma \in {\mathbb R}^{{\tilde{d}}- d}$ below are indexed from $d+ 1$ to ${\tilde{d}}$. All of these are defined at a specific iteration *n*.

#### Lemma 3.2

Let $n \in \{1, \dots , \ell \}$ and define the matrices $B, C, D \in {\mathbb R}^{({\tilde{d}}- d) \times ({\tilde{d}}- d)}$ as

16 $$\begin{aligned} B = {{\,\textrm{diag}\,}}(b_{d+ 1}, \dots , b_{\tilde{d}}),  &   C_{ij} = \zeta _{n - 1}^{j}(\lambda _{i}),  &   D = {{\,\textrm{diag}\,}}\!\Big (\Vert \zeta _{n - 1}^{d+ 1}\Vert ^2, \dots , \Vert \zeta _{n - 1}^{{\tilde{d}}}\Vert ^2\Big ) \end{aligned}$$

for $i, j \in \{d+ 1, \dots , {\tilde{d}}\}$. Also define the vector $w = \big (\pi _n(\lambda _{d+ 1}), \dots , \pi _n(\lambda _{\tilde{d}})\big )^\top$. Then the matrix $D + B^2C$ is invertible and

17 $$\begin{aligned} \tilde{\pi }_n = \pi _n - \sum _{j = d+ 1}^{\tilde{d}}\sigma _j \zeta _{n - 1}^j, \end{aligned}$$

where $$\sigma = (D + B^2C)^{-1}B^2w$$.

#### Proof

Given $j \in \{d+ 1, \dots , {\tilde{d}}\}$, define the polynomial

18 $$\begin{aligned} p_j = \sum _{i = 0}^{n - 1}\frac{\tilde{\pi }_i(\lambda _j)}{\Vert \tilde{\pi }_i\Vert _\sim ^2}\tilde{\pi }_i. \end{aligned}$$

Decompose the polynomial $\pi _n$ in the orthogonal basis $\{\tilde{\pi }_0, \dots , \tilde{\pi }_n\}$ using Lemma 2.5 (instantiated with the inner product $\langle \cdot , \cdot \rangle _\sim$ rather than $\langle \cdot , \cdot \rangle$). This yields

$$\begin{aligned} \pi _n = \tilde{\pi }_n + \sum _{i = 0}^{n - 1} \frac{\langle \pi _n, \tilde{\pi }_i\rangle _\sim }{\Vert \tilde{\pi }_i\Vert _\sim ^2}\tilde{\pi }_i = \tilde{\pi }_n + \sum _{i = 0}^{n - 1} \frac{\tilde{\pi }_i}{\Vert \tilde{\pi }_i\Vert _\sim ^2}\left( \langle \pi _n, \tilde{\pi }_i\rangle + \sum _{j = d+ 1}^{\tilde{d}}b_j^2 \pi _n(\lambda _j) \tilde{\pi }_i(\lambda _j)\right) , \end{aligned}$$

where the second equality comes from the definition (14) of the inner product $\langle \cdot , \cdot \rangle _\sim$. Observe that $\langle \pi _n, \tilde{\pi }_i\rangle = 0$ for $i \in \{0, \dots , n - 1\}$ because of the characterization of $\pi _n$ in Lemma 2.6. This leads to

19 $$\begin{aligned} \pi _n = \tilde{\pi }_n + \sum _{j = d+ 1}^{\tilde{d}}b_j^2\pi _n(\lambda _j)p_j \end{aligned}$$

with the polynomials $p_j$ from (18). Now let $k \in \{d+ 1, \dots , {\tilde{d}}\}$ and decompose the polynomial $\zeta _{n - 1}^k$ in the orthogonal basis $\{\tilde{\pi }_0, \dots , \tilde{\pi }_{n - 1}\}$ to obtain

$$\begin{aligned} \zeta _{n - 1}^k = \sum _{i = 0}^{n - 1} \frac{\langle \zeta _{n - 1}^k, \tilde{\pi }_i\rangle _\sim }{\Vert \tilde{\pi }_i\Vert _\sim ^2}\tilde{\pi }_i = \sum _{i = 0}^{n - 1} \frac{\tilde{\pi }_i}{\Vert \tilde{\pi }_i\Vert _\sim ^2} \left( \langle \zeta _{n - 1}^k, \tilde{\pi }_i\rangle + \sum _{j = d+ 1}^{\tilde{d}}b_j^2 \zeta _{n - 1}^k(\lambda _j) \tilde{\pi }_i(\lambda _j) \right) . \end{aligned}$$

Combining this with the characterization of $\zeta _{n - 1}^k$ in Lemma 2.9 gives

20 $$\begin{aligned} \zeta _{n - 1}^k = \Vert \zeta _{n - 1}^k\Vert ^2 p_k + \sum _{j = d+ 1}^{\tilde{d}}b_j^2 \zeta _{n - 1}^k(\lambda _j) p_j. \end{aligned}$$

Collecting the identities (19) and (20) in matrix form yields

21 $$\begin{aligned} \tilde{\pi }_n = \pi _n - \begin{bmatrix} p_{d+ 1}&\dots&p_{\tilde{d}}\end{bmatrix} B^2 w \qquad {\text {and}} \qquad \begin{bmatrix} \zeta _{n - 1}^{d+ 1}&\dots&\zeta _{n - 1}^{\tilde{d}}\end{bmatrix} = \begin{bmatrix} p_{d+ 1}&\dots&p_{\tilde{d}}\end{bmatrix}(D + B^2C), \end{aligned}$$

where *B*, *C*, *D* and *w* are defined around (16).

We now show that $M = D + B^2C$ is invertible. First, notice that for all $j \in \{d+ 1, \dots , {\tilde{d}}\}$, the quantity $\Vert \zeta _{n - 1}^j\Vert ^2$ is positive because $n - 1 < \ell$. It follows that *D* is positive definite. Let *G* be the symmetric $({\tilde{d}}- d) \times ({\tilde{d}}- d)$ Gram matrix defined as $G_{ij} = \langle \zeta _{n - 1}^i, \zeta _{n - 1}^j\rangle$. The characterizations of the polynomials $\zeta _{n - 1}^i$ and $\zeta _{n - 1}^j$ in (9) give $G_{ij} = \zeta _{n - 1}^j(\lambda _i) \Vert \zeta _{n - 1}^i\Vert ^2$, or equivalently, $C = D^{-1}G$. According to the Woodbury formula, $M = D + B \cdot (BC)$ is invertible if the matrix $I + B C D^{-1} B = I + B D^{-1} G D^{-1} B$ is invertible. It is the case because the second term is positive semidefinite. Finally, substituting the polynomials $p_{d+ 1}, \dots , p_{\tilde{d}}$ in the first equality of (21) by those of the second equality gives the result. $\square$

**Controlling the CG polynomials.** We now leverage the identity (17) in Lemma 3.2 to obtain relationships between the iterates of CG for the inputs $(\tilde{A}, \tilde{b})$ and $(A, b)$. To this end, we assume from now on that

$$\begin{aligned} \lambda _1 \ge \dots \ge \lambda _{\tilde{d}}, \qquad \qquad \lambda _d> 0 \qquad \qquad {\text {and}} \qquad \qquad \lambda _d> \lambda _{d+ 1}. \end{aligned}$$

(We order the eigenvalues, and suppose that $A\succ 0$ and that there is an eigenvalue gap.) As a result, the roots of $\pi _n$ and $\zeta _{n - 1}^j$ are all positive for $n \in \{1, \dots , \ell \}$ (Lemma 2.3 and Lemma 2.11). This ensures in particular that the polynomials $\varsigma _n = \pi _n / \pi _n(0)$ are well defined (recall from Sect. 2.1 that these polynomials determine the iterates of CG). In order to understand the *n*th iterate of CG on $(\tilde{A}, \tilde{b})$, we proceed in two steps: *(i)* we ensure that the iterate is indeed well defined, meaning that the roots of $\tilde{\pi }_n$ are positive, and *(ii)* we relate the polynomial $\tilde{\varsigma }_n = \tilde{\pi }_n / \tilde{\pi }_n(0)$ to $\varsigma _n$.

To do this, we first rewrite the identity (17) in a way that is more comfortable to manipulate the aforementioned rescaled polynomials $\varsigma _n$ and $\tilde{\varsigma }_n$. We introduce

22 $$\begin{aligned} \xi _n^j = \frac{\zeta _n^j}{\zeta _n^j(0)} \quad {\text {for all }}\, n \in \{0, \dots , \ell - 1\} \,{\text { and }}\, j \in \{d+ 1, \dots , {\tilde{d}}\}, \end{aligned}$$

where the polynomials $\zeta _n^j$ are as in Definition 3.1. Remember that Table 2 summarizes the relationships between all the polynomials defined above. (Again, for convenience, $B, C, D \in {\mathbb R}^{({\tilde{d}}- d) \times ({\tilde{d}}- d)}$ and $w, \sigma \in {\mathbb R}^{{\tilde{d}}- d}$ below are indexed from $d+ 1$ to ${\tilde{d}}$. They also depend on the iteration *n*.)

#### Lemma 3.3

Suppose that $\lambda _d> 0$ and $\lambda _d> \lambda _{d+ 1}$. Let $n \in \{1, \dots , \ell \}$ and define the matrices

$$\begin{aligned} B = {{\,\textrm{diag}\,}}(b_{d+ 1}, \dots , b_{\tilde{d}}),  &   C_{ij} = \xi _{n - 1}^{j}(\lambda _{i}),  &   D = {{\,\textrm{diag}\,}}\!\left( \frac{\Vert \xi _{n - 1}^{d+ 1}\Vert ^2}{\xi _{n - 1}^{d+ 1}(\lambda _{d+ 1})}, \dots , \frac{\Vert \xi _{n - 1}^{{\tilde{d}}}\Vert ^2}{\xi _{n - 1}^{\tilde{d}}(\lambda _{\tilde{d}})}\right) , \end{aligned}$$

for $i, j \in \{d+ 1, \dots , {\tilde{d}}\}$, and where $\xi _{n - 1}^{d + j}$ are as in (22). Also define $w = \big (\varsigma _n(\lambda _{d+ 1}), \dots , \varsigma _n(\lambda _{\tilde{d}})\big )^\top$. With $\sigma = (D + B^2C)^{-1}B^2w$, we have

23 $$\begin{aligned} \tilde{\pi }_n = \pi _n - \pi _n(0) \sum _{j = d+ 1}^{\tilde{d}}\sigma _j \xi _{n - 1}^j. \end{aligned}$$

#### Proof

This is just a rescaled variant of (17), made possible by the assumptions on $\lambda _d$ and $\lambda _{d+1}$. Notice that *B* is unchanged but *C*, *D*, *w* and $\sigma$ are all rescaled. Let $\hat{C}, \hat{D}, \hat{w}$ and $\hat{\sigma }$ denote their counterparts in Lemma 3.2. Then $\hat{C} = C S$ where $S = {{\,\textrm{diag}\,}}\!\big (\zeta _{n - 1}^{d+ 1}(0), \dots , \zeta _{n - 1}^{\tilde{d}}(0)\big )$ is a scaling matrix. We leverage now that $\xi _{n - 1}^j(\lambda _j) = 1/\zeta _{n - 1}^j(0)$ and $\Vert \xi _{n - 1}^j\Vert ^2/\xi _{n - 1}^j(\lambda _j) = \Vert \zeta _{n - 1}^j\Vert ^2/\zeta _{n - 1}^j(0)$ for all $j \in \{d+ 1, \dots , {\tilde{d}}\}$. This yields $\hat{D} = D S$. Finally, $\hat{w} = \pi _n(0) w$, and combining the above relations gives $\hat{\sigma }= \pi _n(0) S^{-1} \sigma$. Plugging it in (17) leads to identity (23). $\square$

For $n \le \ell$, the *n*th iterate of CG on $(\tilde{A}, \tilde{b})$ is well defined if (and only if) the roots of $\tilde{\pi }_n$ are all positive (see Sect. 2.1). Consequently, we now study the roots of $\tilde{\pi }_n$. We prove that they are close to the roots of $\pi _n$ when the entries of $\sigma$ (defined in Lemma 3.3) are small. For this, given a root *z* of $\tilde{\pi }_n$, we exhibit a root $\gamma$ of $\pi _n$ such that the ratio $z/\gamma$ is close to 1. The key is that the roots of the polynomials $\xi _{n - 1}$ and $\pi _n$ interlace (by (22) and Lemma 2.11). This will yield a lower-bound on the roots of $\tilde{\pi }_n$.

**Fig. 4:**
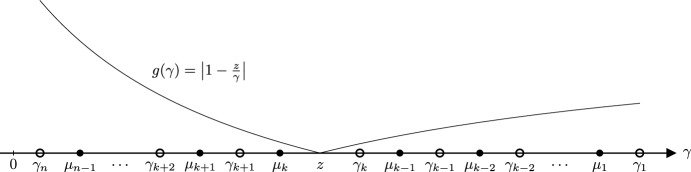
This figure supports the proof of Lemma 3.4. The point *z* may be anywhere between $\gamma _{k + 1}$ and $\gamma _{k - 1}$. Here, it is represented between $\mu _k$ and $\gamma _k$, but the proof considers all scenarios

#### Lemma 3.4

Suppose that $\lambda _d> 0$ and $\lambda _d> \lambda _{d+ 1}$. Let $\gamma _1> \dots > \gamma _n$ be the roots of $\pi _n$ with $n \le \ell$. If *z* is a root of $\tilde{\pi }_n$ then

$$\begin{aligned} \min _{i = 1, \dots , n} \Big |1 - \frac{z}{\gamma _i}\Big | \le \Vert \sigma \Vert _1, \end{aligned}$$

where $\sigma$ (which depends on *n*) is as in Lemma 3.3, and $\Vert \sigma \Vert _1 = |\sigma _{d+1}| + \cdots + |\sigma _{{\tilde{d}}}|$ is its 1-norm.

#### Proof

If $z \in \{\gamma _1, \dots , \gamma _n\}$ then the result is clear. Otherwise, define the function $g :\gamma \mapsto |1 - \frac{z}{\gamma }|$ and let $k \in \mathop {\mathrm {arg\,min}}\limits _{i = 1, \dots , n} g(\gamma _i)$. Let $\mu _1^j> \dots > \mu _{n - 1}^j$ denote the roots of $\xi _{n - 1}^j$. Identity (23) yields

$$\begin{aligned} 0 = \tilde{\pi }_n(z) = \prod _{i = 1}^n (z - \gamma _i) - \pi _n(0)\sum _{j = d+ 1}^{\tilde{d}}\sigma _j \prod _{i = 1}^{n - 1} \bigg (1 - \frac{z}{\mu _i^j} \bigg ). \end{aligned}$$

Divide by $-\gamma _k \prod _{i \ne k} (z - \gamma _i)$ and apply the triangle inequality to obtain

$$\begin{aligned} g(\gamma _k) \le \sum _{j = d+ 1}^{\tilde{d}}|\sigma _j| h_j \qquad \qquad {\text {where}} \qquad \qquad h_j = \frac{\prod _{i = 1}^{n - 1} g(\mu _i^j)}{\prod _{i \ne k} g(\gamma _i)}. \end{aligned}$$

We now let $j \in \{d+ 1, \dots , {\tilde{d}}\}$ and show that $h_j \le 1$. This is clear when $z = 0$ so we consider $z \ne 0$. Under our assumptions, the roots of $\pi _n$ and $\xi _{n - 1}^j$ interlace and are positive (as shown in Lemmas 2.3 and 2.11). First assume that $z < 0$. In this case the function *g* is decreasing on  and it follows that $k = 1$. Together with the interlacement of the roots, this implies that $h_j \le 1$. Now assume that $z > 0$. The function *g* is decreasing on  and increasing on . This implies that $\gamma _{k + 1}< z < \gamma _{k - 1}$ (we set $\gamma _0 = +\infty$ and $\gamma _{n + 1} = 0$). We obtain the following pattern for the roots:

24 $$\begin{aligned} \begin{aligned} 0<\gamma _n< \mu _{n - 1}^j< \dots< \gamma _{k + 2}< \mu _{k + 1}^j<&\gamma _{k + 1}< z< \gamma _{k - 1}< \mu _{k - 2}^j< \gamma _{k - 2}< \dots< \mu _1^j< \gamma _1\\ {\text {and}} \qquad \qquad&\gamma _{k + 1}< \mu _k^j< \gamma _k< \mu _{k - 1}^j < \gamma _{k - 1}. \end{aligned} \end{aligned}$$

Figure 4 is an illustration of this interlacement and of *g*. Observe from (24) that $g(\gamma _i) \ge g(\mu _{i - 1}^j)$ for all $i \in \{n, \dots , k + 2\}$ because *g* is decreasing on . Likewise, we have $g(\mu _i^j) \le g(\gamma _i)$ for all $i \in \{k - 2, \dots , 1\}$ because *g* is increasing on . From the monotonicity pattern of *g* we deduce that $g(\mu _k^j) \le \max (g(\gamma _{k + 1}), g(\gamma _k))$. To see this observe that it holds whether ,  or $z \ge \gamma _k$. The minimality of $\gamma _k$ then implies that $g(\mu _k^j) \le g(\gamma _{k + 1})$. Likewise, $g(\mu _{k - 1}^j) \le \max (g(\gamma _k), g(\gamma _{k - 1})) = g(\gamma _{k - 1})$. Combining all the previous inequalities yields $h_j \le 1$. $\square$

If we additionally assume $\Vert \sigma \Vert _1 < 1$, we have a useful corollary.

#### Corollary 3.5

Suppose that $\lambda _d> 0$ and $\lambda _d> \lambda _{d+ 1}$. If $\Vert \sigma \Vert _1 < 1$ for some $n \le \ell$, then the roots of $\tilde{\pi }_n$ are lower-bounded by $\big (1 - \Vert \sigma \Vert _1\big ) \gamma _n$. In particular, they are positive.

#### Proof

Let *z* be a root of $\tilde{\pi }_n$ and $k \in \mathop {\mathrm {arg\,min}}\limits _{i = 1, \dots , n} \big |1 - \frac{z}{\gamma _i}\big |$. Lemma 3.4 gives that $\gamma _k - z \le \gamma _k \Vert \sigma \Vert _1$. Rearranging the terms gives the result, using that $\gamma _k \ge \gamma _n > 0$. $\square$

We now combine all the results above to obtain *(i)* a sufficient condition for the *n*th iteration of CG on $(\tilde{A}, \tilde{b})$ to be well defined, and, when it is the case, *(ii)* a relationship between $\tilde{\varsigma }_n$ and $\varsigma _n$. Here we let $\textbf{1}$ denote the vector of all ones.

#### Theorem 3.6

Suppose that $\lambda _d> 0$ and $\lambda _d> \lambda _{d+ 1}$. Let $n \in \{1, \dots , \ell \}$ and $\sigma$ as in Lemma 3.3. If $\Vert \sigma \Vert _1 < 1$ then the *n*th iteration of CG on $(\tilde{A}, \tilde{b})$ is well defined, and the associated polynomial $\tilde{\varsigma }_n = \tilde{\pi }_n/\tilde{\pi }_n(0)$ satisfies

25 $$\begin{aligned} \tilde{\varsigma }_n = \frac{1}{1 - s}\bigg ( \varsigma _n - \sum _{j = d+ 1}^{\tilde{d}}\sigma _j \xi _{n - 1}^j \bigg ), \qquad \qquad {\text {where}} \qquad \qquad s = \textbf{1}^\top \sigma . \end{aligned}$$

#### Proof

Corollary 3.5 gives that the roots of $\tilde{\pi }_n$ are positive. Lemma 2.7 then implies that the matrix  is positive definite, where ${\tilde{\mathcal {K}}}_n$ is the *n*th Krylov subspace associated to $(\tilde{A}, \tilde{b})$. Thus, the *n*th iteration of CG on $(\tilde{A}, \tilde{b})$ is well defined. The definition of $\tilde{\varsigma }_n$ and the identity (23) provide

$$\begin{aligned} \tilde{\varsigma }_n = \frac{\tilde{\pi }_n}{\tilde{\pi }_n(0)} = \frac{\pi _n - \pi _n(0) \sum _{j = d+ 1}^{\tilde{d}}\sigma _j \xi _{n - 1}^j}{\pi _n(0) - \pi _n(0) \sum _{j = d+ 1}^{\tilde{d}}\sigma _j} = \frac{\varsigma _n - \sum _{j = d+ 1}^{\tilde{d}}\sigma _j \xi _{n - 1}^j}{1 - \sum _{j = d+ 1}^{\tilde{d}}\sigma _j}, \end{aligned}$$

which is the intended equality. $\square$

**From the polynomials to the iterates.** Theorem 3.6 explicitly links the polynomials $\varsigma _n$ and $\tilde{\varsigma }_n$, associated to the problems $(A, b)$ and $(\tilde{A}, \tilde{b})$ respectively. These polynomials determine the iterates of CG (see Sect. 2.1). Indeed, let $\varphi _{n - 1}$ and $\tilde{\varphi }_{n - 1}$ be the polynomials that satisfy $\varsigma _n(x) = 1 - x \varphi _{n - 1}(x)$ and $\tilde{\varsigma }_n(x) = 1 - x \tilde{\varphi }_{n - 1}(x)$ respectively. Then $v_n = \varphi _{n - 1}(A) b$ and $\tilde{v}_n = \tilde{\varphi }_{n - 1}(\tilde{A}) \tilde{b}$. We exploit this to relate the iterates on the two problems. First, we need two helper lemmas to control $\varsigma _n - \xi _{n - 1}^j$.

#### Lemma 3.7

Suppose that $\lambda _d> 0$ and $\lambda _d> \lambda _{d+ 1}$. Let $n \in \{1, \dots , \ell \}$ and $j \in \{d+ 1, \dots , {\tilde{d}}\}$. Define the polynomials $p_0, \ldots , p_{n - 1}$ as

$$\begin{aligned} p_k(x) = \frac{\varsigma _n(x) - \xi _k^j(x)}{x}. \end{aligned}$$

Then, $$\Vert p_{n - 1}\Vert \le \cdots \le \Vert p_1\Vert \le \Vert p_0\Vert = \Vert \varphi _{n - 1}\Vert = \Vert v_n\Vert $$.

#### Proof

We fix *j* and omit for all *k* the superscript of $\xi _k^j$ for conciseness. We let $k \in \{0, \dots , n - 2\}$ and prove that $\Vert p_{k + 1}\Vert \le \Vert p_k\Vert$. We can rewrite identity (10) as

$$\begin{aligned} \frac{\xi _{k + 1}(\lambda _j)}{\Vert \xi _{k + 1}\Vert ^2} \xi _{k + 1} = \frac{\xi _k(\lambda _j)}{\Vert \xi _k\Vert ^2} \xi _k + \frac{\varsigma _{k + 1}(\lambda _j)}{\Vert \varsigma _{k + 1}\Vert ^2} \varsigma _{k + 1} \end{aligned}$$

using the fact that $\xi _k(\lambda _j) = 1/\zeta _k^j(0)$ and $\xi _{k + 1}(\lambda _j) = 1/\zeta _{k + 1}^j(0)$. The roots of $\xi _k$, $\xi _{k + 1}$ and $\varsigma _{k + 1}$ are all in  (Lemmas 2.3 and 2.11) and $\xi _k(0) = \xi _{k + 1}(0) = \varsigma _{k + 1}(0) = 1$. We deduce that all the coefficients in the identity above are (strictly) positive. Divide by $\xi _k(\lambda _j)/\Vert \xi _k\Vert ^2$ and leverage again $\xi _k(0) = \xi _{k + 1}(0) = \varsigma _{k + 1}(0) = 1$ to obtain that

26 $$\begin{aligned} \xi _k = (1 + \alpha ) \xi _{k + 1} - \alpha \varsigma _{k + 1} \end{aligned}$$

for some number $\alpha \ge 0$. It follows that $p_k = p_{k + 1} + \alpha q_{k + 1}$, where we define the polynomial

$$\begin{aligned} q_{k + 1}(x) = \frac{\varsigma _{k + 1}(x) - \xi _{k + 1}(x)}{x}. \end{aligned}$$

In order to prove that $\Vert p_{k + 1}\Vert ^2 \le \Vert p_k\Vert ^2$, it is sufficient to show that the inner product $\langle p_{k + 1}, q_{k + 1}\rangle$ is non-negative. We can rewrite

$$\begin{aligned} \langle p_{k + 1}, q_{k + 1}\rangle = \Vert q_{k + 1}\Vert ^2 + \langle p_{k + 1} - q_{k + 1}, q_{k + 1}\rangle . \end{aligned}$$

The first term is positive; let us prove the second term is non-negative. A Taylor expansion yields

$$\begin{aligned} x q_{k + 1}(x) = \varsigma _{k + 1}(x) - \xi _{k + 1}(x) = \big (\varsigma _{k + 1}'(0) - \xi _{k + 1}'(0)\big ) x + x^2 h(x) \end{aligned}$$

for all *x*, where *h* is some polynomial of degree at most $k - 1$. It follows that

$$\begin{aligned} \langle p_{k + 1} - q_{k + 1}, q_{k + 1}\rangle&= \sum _{i = 1}^d\big (\varsigma _n(\lambda _i) - \varsigma _{k + 1}(\lambda _i)\big )\bigg (\frac{\varsigma _{k + 1}'(0) - \xi _{k + 1}'(0)}{\lambda _i} + h(\lambda _i)\bigg ) b_i^2\\&= \big (\varsigma _{k + 1}'(0) - \xi _{k + 1}'(0)\big ) \sum _{i = 1}^d\frac{\varsigma _n(\lambda _i) - \varsigma _{k + 1}(\lambda _i)}{\lambda _i} b_i^2 \end{aligned}$$

because $\varsigma _n$ and $\varsigma _{k + 1}$ are both orthogonal to *h* for the inner product $\langle \cdot , \cdot \rangle$ (Lemma 2.6). From (26) we find that $\varsigma _{k + 1}'(0) - \xi _{k + 1}'(0) = (1 + \alpha )^{-1}\big (\varsigma _{k + 1}'(0) - \xi _k'(0)\big )$. Let $\gamma _1> \dots > \gamma _{k + 1}$ and $\mu _1> \dots > \mu _k$ be the roots of $\varsigma _{k + 1}$ and $\xi _k$ respectively. Notice that

$$\begin{aligned} \varsigma _{k + 1}'(0) = -\sum _{i = 1}^{k + 1} \frac{1}{\gamma _i}< \xi _k'(0) = -\sum _{i = 1}^k \frac{1}{\mu _i} < 0, \end{aligned}$$

where the inequalities are because the roots of these polynomials interlace and are all positive (Lemmas 2.3 and 2.11). We deduce that $\varsigma _{k + 1}'(0) - \xi _{k + 1}'(0)$ is negative. Finally, notice that $\varsigma _n$ and $\varsigma _{k + 1}$ are orthogonal to the polynomials $x \mapsto \big (1 - \varsigma _n(x)\big )/x$ and $x \mapsto \big (1 - \varsigma _{k + 1}(x)\big )/x$ respectively (Lemma 2.6). This implies that

$$\begin{aligned} \sum _{i = 1}^d\frac{\varsigma _n(\lambda _i)}{\lambda _i} b_i^2 = \sum _{i = 1}^d\frac{\varsigma _n(\lambda _i)^2}{\lambda _i} b_i^2 \qquad \qquad {\text {and}} \qquad \qquad \sum _{i = 1}^d\frac{\varsigma _{k + 1}(\lambda _i)}{\lambda _i} b_i^2 = \sum _{i = 1}^d\frac{\varsigma _{k + 1}(\lambda _i)^2}{\lambda _i} b_i^2. \end{aligned}$$

Combine this with the minimization property of $\varsigma _n$ given in Lemma 2.8 to conclude that the inner product $\langle p_{k + 1} - q_{k + 1}, q_{k + 1}\rangle$ is non-negative. $\square$

#### Lemma 3.8

Suppose that $\lambda _d> 0$ and $\lambda _d> \lambda _{d+ 1}$. Let $n \in \{1, \dots , \ell \}$ and $j \in \{d+ 1, \dots , {\tilde{d}}\}$. Then $\big |\varsigma _n(x) - \xi _{n - 1}^j(x)\big | \le |\varsigma _n(x) - 1|$ for all $x \le \lambda _d$.

#### Proof

Let $\gamma _1> \dots > \gamma _n$ and $\mu _1> \dots > \mu _{n - 1}$ be the roots of $\varsigma _n$ and $\xi _{n - 1}^j$ respectively. Then we can write

$$\begin{aligned} \varsigma _n(x) = \prod _{i = 1}^n \bigg (1 - \frac{x}{\gamma _i}\bigg ) \qquad {\text {and}} \qquad \xi _{n - 1}^j(x) = \prod _{i = 1}^{n - 1} \bigg (1 - \frac{x}{\mu _i}\bigg ) \end{aligned}$$

for all $x \in {\mathbb R}$. The roots of $\varsigma _n$ and $\xi _{n - 1}^j$ interlace, and are all at least $\lambda _d$ (Lemmas 2.3 and 2.11). We deduce from these properties and from the expressions above that $\varsigma _n(x) \ge \xi _{n - 1}^j(x) \ge 1$ for all $x \le 0$ and $1 \ge \xi _{n - 1}^j(x) \ge \varsigma _n(x) \ge 0$ for all . The claim holds in both cases. $\square$

We now compare the iterates $v$ and $\tilde{v}$ of CG on $(A, b)$ and $(\tilde{A}, \tilde{b})$ at a given iteration. The following bounds show that, in a certain regime, the first $d$ components of $\tilde{v}$ are close to $v$, and the last ${\tilde{d}}- d$ components are close to zero. This means that (in this regime) CG on $(\tilde{A}, \tilde{b})$ essentially ignores the eigenvalues $\lambda _{d+ 1\phantom {{\tilde{d}}}}, \dots , \lambda _{{\tilde{d}}}$ and the weights $b_{d+ 1\phantom {{\tilde{d}}}}, \dots , b_{\tilde{d}}$.

#### Theorem 3.9

Suppose that $\lambda _d> 0$ and $\lambda _d> \lambda _{d+ 1}$. Let $n \in \{1, \dots , \ell \}$ and $\sigma$ as in Lemma 3.3. Suppose that $\Vert \sigma \Vert _1 < 1$ so that the *n*th iteration of CG on $(\tilde{A}, \tilde{b})$ is well defined (Theorem 3.6). Let $v$ and $\tilde{v}$ denote the *n*th iterates of CG on $(A, b)$ and $(\tilde{A}, \tilde{b})$ respectively. Then

$$\begin{aligned} \Vert \tilde{v}_{1:d} - v\Vert \le \frac{\Vert \sigma \Vert _1}{1 - \Vert \sigma \Vert _1}\Vert v\Vert \qquad {\text {and}} \qquad |\tilde{v}_i| \le \frac{|b_i|}{1 - \Vert \sigma \Vert _1} \bigg | \frac{\big (1 - \lambda _i/\lambda _d\big )^n - 1}{\lambda _i} \bigg | \end{aligned}$$

for all $i \in \{d+ 1, \dots , {\tilde{d}}\}$, where $\tilde{v}_{1:d}$ extracts the first $d$ entries of $\tilde{v}$. (The absolute value in the expression on the right evaluates to $n/\lambda _d$ in the limit where $\lambda _i = 0$.)

#### Proof

Define the polynomials

$$\begin{aligned}&\textstyle \varphi _{n - 1}(x) = \frac{1 - \varsigma _n(x)}{x}, \textstyle \tilde{\varphi }_{n - 1}(x) = \frac{1 - \tilde{\varsigma }_n(x)}{x} \\&{\text {and}} \,\,p_{n - 1}^j(x) = \frac{\varsigma _n(x) - \xi _{n - 1}^j(x)}{x}. \end{aligned}$$

The iterates satisfy $v= \varphi _{n - 1}(A)b$ and $\tilde{v}= \tilde{\varphi }_{n - 1}(\tilde{A})\tilde{b}$ as recalled in Sect. 2.1. We exploit the link given in Theorem 3.6 to relate $v$ and $\tilde{v}$. Rearranging the terms in (25) gives

27 $$\begin{aligned} \tilde{\varsigma }_n - \varsigma _n = \frac{1}{1 - s} \sum _{j = d+ 1}^{\tilde{d}}\sigma _j\big (\varsigma _n - \xi _{n - 1}^j\big ) \qquad {\text {where}} \qquad s = \textbf{1}^\top \sigma . \end{aligned}$$

For all $i \in \{1, \dots , d\}$ we have $\tilde{v}_i - v_i = \big (\tilde{\varphi }_{n - 1}(\lambda _i) - \varphi _{n - 1}(\lambda _i)\big ) b_i$. Combining this with (27) gives

$$\begin{aligned} \Vert \tilde{v}_{1:d} - v\Vert ^2&= \frac{1}{(1 - s)^2} \sum _{i = 1}^d\bigg ( \sum _{j = d+ 1}^{\tilde{d}}\sigma _j p_{n - 1}^j(\lambda _i) b_i \bigg )^2 \\&= \frac{1}{(1 - s)^2} \sum _{j = d+ 1}^{\tilde{d}}\sum _{k = d+ 1}^{\tilde{d}}\sigma _j\sigma _k \langle p_{n - 1}^j, p_{n - 1}^k\rangle , \end{aligned}$$

where $p_{n - 1}^j$ is the polynomial we defined above. Lemma 3.7 and the Cauchy–Schwarz inequality imply that $|\langle p_{n - 1}^j, p_{n - 1}^k\rangle | \le \Vert v\Vert ^2$ for all *j*, *k*. Combine this with the triangle inequality to obtain $\Vert \tilde{v}_{1:d} - v\Vert ^2 \le (1 - s)^{-2} \Vert \sigma \Vert _1^2 \Vert v\Vert ^2$. Now let $i \in \{d+ 1, \dots , {\tilde{d}}\}$. From (27) we compute

$$\begin{aligned} \tilde{\varphi }_{n - 1}(\lambda _i) = \frac{1}{\lambda _i}\bigg ( 1 - \varsigma _n(\lambda _i) + \frac{1}{1 - s} \sum _{j = d+ 1}^{\tilde{d}}\sigma _j \big ( \xi _{n - 1}^j(\lambda _i) - \varsigma _n(\lambda _i) \big )\bigg ). \end{aligned}$$

Then, by the triangle inequality and Lemma 3.8, $|\tilde{\varphi }_{n - 1}(\lambda _i)| \le |\lambda _i|^{-1} |\varsigma _n(\lambda _i) - 1| \big ( 1 + (1 - s)^{-1} \Vert \sigma \Vert _1 \big )$. The polynomial $\varsigma _n$ has *n* real roots $\gamma _1 \ge \cdots \ge \gamma _n$, with $\gamma _n \ge \lambda _d$ (Lemma 2.3). Thus, $\varsigma _n(\lambda _i) = (1-\lambda _i/\gamma _1) \cdots (1-\lambda _i/\gamma _n) \ge 0$ (each factor is positive since $\lambda _i < \lambda _d$ and $\lambda _d> 0$). If $\lambda _i \le 0$, then $1 \le \varsigma _n(\lambda _i) \le (1 - \lambda _i/\lambda _d)^n$. If $\lambda _i \ge 0$, then $1 \ge \varsigma _n(\lambda _i) \ge (1 - \lambda _i/\lambda _d)^n$. In all cases, $|\varsigma _n(\lambda _i) - 1| \le |(1 - \lambda _i/\lambda _d)^n - 1|$. This gives the bound on $|\tilde{v}_i|$. $\square$

Note that the bound $\Vert v\Vert \le \lambda _d^{-1} \Vert b\Vert$ always holds when $\lambda _d> 0$, because for $A\succ 0$ all iterates are smaller than the solution $A^{-1}b$ (see references in Lemma 4.4). Thus, the results above imply in particular $\Vert \tilde{v}_{1:d} - v\Vert \le \lambda _d^{-1}(1 - \Vert \sigma \Vert _1)^{-1}\Vert \sigma \Vert _1\Vert b\Vert$.

### Implications for our regime of interest

The important hypothesis in Theorems 3.6 and 3.9 is that the entries of $\sigma$ are small. In this section, we show that this is indeed the case for the regime that will matter in the local convergence analysis of trust-region methods in Sect. 4. Specifically, we assume that

28 $$\begin{aligned} |\lambda _{d+ 1}|, \dots , |\lambda _{\tilde{d}}| \ll \lambda _d\qquad {\text {and}} \qquad b_{d+1}^2 + \cdots + b_{\tilde{d}}^2 \ll b_1^2 + \cdots + b_d^2. \end{aligned}$$

That is, the last ${\tilde{d}}- d$ eigenvalues of $\tilde{A}$ are close to zero, and the last ${\tilde{d}}- d$ components of $\tilde{b}$ carry little weight. Under these conditions, the matrix *C* (defined in Lemma 3.3) is close to the rank one matrix $\textbf{1}\textbf{1}^\top$ and we can control the entries of $\sigma$.

The polynomials $\xi _{n - 1}$ and $\varsigma _n$ satisfy $\xi _{n - 1}(0) = \varsigma _n(0) = 1$ so the values $\xi _{n - 1}(x)$ and $\varsigma _n(x)$ are close to one when *x* is close to zero. To quantify this, we define

29 $$\begin{aligned} \delta = \max _{i, j \in \{d+ 1, \dots , {\tilde{d}}\}} \big |\xi _{n - 1}^j(\lambda _{i}) - 1\big | \qquad {\text {and}} \qquad \tau = \max _{i \in \{d+ 1, \dots , {\tilde{d}}\}} \big |\varsigma _n(\lambda _i) - 1\big |. \end{aligned}$$

Note that these two numbers depend on the iteration *n* but it will always be clear from context. For each coefficient $\sigma _j$, we define below a reference quantity $\beta _j = b_j^2/D_{jj}$ and show $\sigma _j = \beta _j + o(\beta _j)$ in the regime (28) (the matrix *D* comes from Lemma 3.3). We start with a helper lemma.

#### Lemma 3.10

Given $S, M \succeq 0$, the matrix $X = (I + S M)^{-1} S$ satisfies

$$\begin{aligned} \max _{ij} |X_{ij}| \le \max _{ij} |S_{ij}|. \end{aligned}$$

#### Proof

We can factorize $M = K K^\top$ for some matrix *K*. The Woodbury formula provides

$$\begin{aligned} X = \bigg ( I - S K (I + K^\top S K)^{-1} K^\top \bigg ) S = S^{1/2} \bigg ( I - S^{1/2} K (I + K^\top S K)^{-1} K^\top S^{1/2} \bigg ) S^{1/2}. \end{aligned}$$

Let $S^{1/2} K = U \Sigma V^\top$ be a singular value decomposition. We can rewrite the previous identity as

$$\begin{aligned} X = S^{1/2} Y S^{1/2} \qquad {\text {where}} \qquad Y = U (I + \Sigma ^2)^{-1} U^\top . \end{aligned}$$

Let *w* be the column of $S^{1/2}$ of maximal 2-norm. Notice that $\max _{ij} |S_{ij}| = \Vert w\Vert ^2$. Now let *u* and *v* be the *i*th and *j*th columns of $S^{1/2}$ respectively. Then

$$\begin{aligned} |X_{ij}| = |u^\top Y v| \le \Vert u\Vert \Vert v\Vert \le \Vert w\Vert ^2 = \max _{ij} |S_{ij}|, \end{aligned}$$

where the first inequality is because $\Vert Y\Vert \le 1$ and the second inequality is the maximality of *w*. $\square$

#### Lemma 3.11

Under the assumptions (and with the notation) of Lemma 3.3, and with $\delta , \tau$ as in (29), the entries of $\sigma$ satisfy

$$\begin{aligned} \big |\sigma _j - \beta _j\big | \le \Big (\tau + (1 + \tau )(1 + \delta ) \Vert D^{-1}\Vert \Vert B\Vert _\textrm{F}^2 \Big ) \beta _j \qquad {\text {where}} \qquad \beta = D^{-1}B^2. \end{aligned}$$

#### Proof

Apply the Woodbury formula to $\sigma = (D + B^2 \cdot C)^{-1} B^2 w$ to obtain

$$\begin{aligned} \sigma&= D^{-1} B^2 \big (I - X B^2\big ) w \qquad {\text {where}} \qquad X = \big (I + C D^{-1} B^2\big )^{-1} C D^{-1}. \end{aligned}$$

If we let *G* denote the Gram matrix $G_{ij} = \langle \xi _{n - 1}^i, \xi _{n - 1}^j\rangle$ then $C = D^{-1} G$ (in the same way as in the proof of Lemma 3.2). It follows that $C D^{-1} = D^{-1} G D^{-1}$ is positive semidefinite (because *D* is positive definite). By Lemma 3.10 (with $S = C D^{-1}$ and $M = B^2$), it follows that

$$\begin{aligned} \max _{ij} |X_{ij}| \le \max _{ij} \big |(C D^{-1})_{ij}\big | \le (1 + \delta ) \Vert D^{-1}\Vert . \end{aligned}$$

Finally, the *j*th entry of $\sigma$ satisfies $\sigma _j = \beta _j\big (w_j - ( X B^2 w )_j\big )$, hence

$$\begin{aligned} \big |\sigma _j - \beta _j\big | = \beta _j \bigg | (w_j - 1) - \sum _{k = d+ 1}^{{\tilde{d}}} X_{jk} B_{kk}^2 w_k \bigg |, \end{aligned}$$

which we can bound with the triangle inequality and using $|w_k - 1| \le \tau$ for all *k* due to (29). $\square$

#### Corollary 3.12

Under the assumptions (and with the notation) of Lemma 3.3, and with $\delta , \tau$ as in (29), the entries of $\sigma$ satisfy

$$\begin{aligned} |\sigma _j| \le (1 + \tau )\Big (1 + (1 + \delta )\Vert D^{-1}\Vert \Vert B\Vert _\textrm{F}^2\Big ) D_{jj}^{-1} B_{jj}^2. \end{aligned}$$

#### Proof

The triangle inequality gives $|\sigma _j| \le \beta _j + |\sigma _j - \beta _j|$. Conclude with Lemma 3.11. $\square$

The bounds given in Lemma 3.11 and Corollary 3.12 involve the entries of the matrix *D* (defined in Lemma 3.3). We need to control them, and more specifically, to lower-bound the norms $\Vert \xi _{n - 1}^j\Vert$ for $j \in \{d+ 1, \dots , {\tilde{d}}\}$. We now (implicitly) exhibit an iteration for which we can secure such bounds. Remember that $\ell$ denotes the grade of $(A, b)$.

#### Lemma 3.13

We use the assumptions and notation of Lemma 3.3, and $\delta , \tau$ are as in (29). Given a parameter , there exists an iteration $n \in \{1, \dots , \ell \}$ such that $\Vert \varsigma _n\Vert ^2 \le \lambda _1 c$ and $\Vert \xi _{n - 1}^j\Vert ^2 \ge \lambda _dc$ for all $j \in \{d+ 1, \dots , {\tilde{d}}\}$. In particular, this implies $\Vert \sigma \Vert _1 \le \omega$ at iteration *n*, where

$$\begin{aligned} \eta = \tau + (1 + \tau )(1 + \delta )^2 (\lambda _dc)^{-1} \Vert B\Vert _\textrm{F}^2 \qquad {\text {and}} \qquad \omega = (1 + \eta )(1 + \delta )(\lambda _dc)^{-1}\Vert B\Vert _\textrm{F}^2. \end{aligned}$$

#### Proof

Let $n \in \{1, \dots , \ell \}$ be the *smallest* integer such that

30 $$\begin{aligned} \min _{\varsigma \in \mathcal {P}_{\le n}} \; \sum _{j = 1}^d\frac{\varsigma (\lambda _j)^2}{\lambda _j} b_j^2 \le c\qquad {\text {subject to}} \qquad \varsigma (0) = 1. \end{aligned}$$

Such an integer exists because when $n = \ell$ the minimum value of the above optimization problem is zero (by picking a polynomial whose roots are exactly the eigenvalues of $A$ with positive weight, see Remark 2.4). Lemma 2.8 states that $\varsigma _n$ is the solution of the optimization problem (30). From the definition of the semi-norm $\Vert \cdot \Vert$ in (14) it follows that $\Vert \varsigma _n\Vert ^2 \le \lambda _1 c$. Since *n* is the smallest integer with the property above, the cost function of (30) is larger than *c* when evaluated at any feasible polynomial of degree $n-1$ or less. In particular, for all *j* the polynomial $\xi _{n - 1}^j$ is feasible for (30) because $\xi _{n - 1}^j(0) = 1$. Again from the definition of the semi-norm $\Vert \cdot \Vert$ in (14), we deduce that $\Vert \xi _{n - 1}^j\Vert ^2 \ge \lambda _dc$ for all $j \in \{d+ 1, \dots , {\tilde{d}}\}$. (This inequality also holds when $n = 1$ because $\xi _0^j \equiv 1$ and we assumed $c \le \lambda _d^{-1}\Vert b\Vert ^2$.) In particular, it implies that $\Vert D^{-1}\Vert \le (1 + \delta )(\lambda _dc)^{-1}$. Apply Corollary 3.12 to obtain

$$\begin{aligned} \Vert \sigma \Vert _1&\le (1 + \tau )\Big (1 + (1 + \delta )\Vert D^{-1}\Vert \Vert B\Vert _\textrm{F}^2\Big )\sum _{j = d+ 1}^{\tilde{d}}D_{jj}^{-1} B_{jj}^2\\&\le (1 + \eta )(1 + \delta )(\lambda _dc)^{-1}\Vert B\Vert _\textrm{F}^2 = \omega , \end{aligned}$$

where $\eta$ and $\omega$ are defined in this lemma statement. $\square$

Provided the specific iteration *n* from Lemma 3.13 is well defined on the problem $(\tilde{A}, \tilde{b})$, we can derive upper-bounds for the norms of its corresponding iterate and residual. We could obtain such bounds from Theorem 3.9. Here we obtain different ones using the special regime (28).

#### Lemma 3.14

We use the assumptions and notation of Lemma 3.3, and $\delta , \tau$ are as in (29). Given a parameter , let $\eta$ and $\omega$ be as in Lemma 3.13, and assume that $\omega < 1$. Then there exists an integer $n \in \{1, \dots , \ell \}$ such that the *n*th iteration of CG on $(\tilde{A}, \tilde{b})$ is well defined. Moreover, the *n*th iterate $\tilde{v}_n$ and residual $\tilde{r}_n$ satisfy

$$\begin{aligned} \Vert \tilde{v}_n\Vert&\le \frac{1}{(1 - \omega ) \lambda _d} \Vert \tilde{b}\Vert \quad {\text {and}}\\&\quad \Vert \tilde{r}_n\Vert ^2 \le \frac{1}{(1 - \omega )^2}\bigg ( \lambda _1 c+ (1 + \eta )(1 + \delta )\omega \Vert B\Vert _\textrm{F}^2 + ( 1 + \tau )^2 \Vert B\Vert _\textrm{F}^2 \bigg ). \end{aligned}$$

#### Proof

Let *n* be an integer as in Lemma 3.13. Since $\Vert \sigma \Vert _1 \le \omega < 1$, we can apply Theorem 3.6 and deduce that the *n*th iteration of CG on $(\tilde{A}, \tilde{b})$ is well defined. Corollary 3.5 gives that $(1 - \omega ) \lambda _d$ is a lower-bound on the roots of $\tilde{\varsigma }_n$. Let ${\tilde{\mathcal {K}}}_n$ be the *n*th Krylov space associated to $(\tilde{A}, \tilde{b})$. Lemma 2.7 ensures that . As recalled in Sect. 2.1, if we let $Q_n$ be an orthonormal basis of $\tilde{\mathcal {K}}_n$ then we can write the *n*th iterate of CG as

$$\begin{aligned} \tilde{v}_n = Q_n\big (Q_n^\top \tilde{A}Q_n  \big )^{-1}Q_n^\top \tilde{b}. \end{aligned}$$

It follows that $\Vert \tilde{v}_n\Vert \le (1 - \omega )^{-1} \lambda _d^{-1} \Vert \tilde{b}\Vert$. We now bound the norm of the residual. The identity (25) and the definition (14) of $\Vert \cdot \Vert _\sim$ give

$$\begin{aligned}&\Vert \tilde{r}_n\Vert ^2 = \Vert \tilde{\varsigma }_n\Vert _\sim ^2 = \frac{1}{(1 - s)^{2}} \bigg ( \Vert \varsigma _n\Vert ^2 + \Vert p\Vert ^2 + \sum _{i = d+ 1}^{\tilde{d}}\Big (\varsigma _n(\lambda _i) - p(\lambda _i)\Big )^2 b_i^2 \bigg )\\&\qquad {\text {where}}\quad p= \sum _{j = d+ 1}^{\tilde{d}}\sigma _j \xi _{n - 1}^j \end{aligned}$$

and $s = \textbf{1}^\top \sigma$. Here, we used the fact that $\langle \varsigma _n, p\rangle = 0$. We now bound each term separately. First, we have $\Vert \varsigma _n\Vert ^2 \le \lambda _1 c$ (Lemma 3.13). The triangle inequality and Corollary 3.12 give

$$\begin{aligned} \Vert p\Vert&\le \sum _{j = d+ 1}^{\tilde{d}}\Vert \sigma _j \xi _{n - 1}^j\Vert \le (1 + \eta ) \sum _{j = d+ 1}^{\tilde{d}}D_{jj}^{-1} B_{jj}^2 \Vert \xi _{n - 1}^j\Vert \\&= (1 + \eta ) \sum _{j = d+ 1}^{\tilde{d}}\xi _{n - 1}^j(\lambda _j) \Vert \xi _{n - 1}^j\Vert ^{-1} B_{jj}^2, \end{aligned}$$

where we used the definition of *D* in Lemma 3.3 for the last equality. It follows that $\Vert p\Vert ^2 \le (1 + \eta )^2(1 + \delta )^2(\lambda _dc)^{-1}\Vert B\Vert _\textrm{F}^4$. We now consider the third term. For all $i \in \{d+ 1, \dots , {\tilde{d}}\}$ we have

$$\begin{aligned} \varsigma _n(\lambda _i) - p(\lambda _i) = (1 - s)\varsigma _n(\lambda _i) + \sum _{j = d+ 1}^{\tilde{d}}\sigma _j\big ( \varsigma _n(\lambda _i) - \xi _{n - 1}^j(\lambda _i) \big ). \end{aligned}$$

By Lemma 3.8 and since $|s| \le \Vert \sigma \Vert _1 \le \omega < 1$, the above is upper-bounded in absolute value as

$$\begin{aligned} |\varsigma _n(\lambda _i) - p(\lambda _i)|&\le (1-s)\left( 1 + \tau + \frac{\Vert \sigma \Vert _1}{1-s} \tau \right) \le (1-s) \left( 1 + \tau + \frac{\tau \omega }{1 - \omega } \right) \\&\le (1-s) \frac{1 + \tau }{1 - \omega }. \end{aligned}$$

Thus,

$$\begin{aligned} \frac{1}{(1 - s)^2} \sum _{i = d+ 1}^{\tilde{d}}\Big (\varsigma _n(\lambda _i) - p(\lambda _i)\Big )^2 b_i^2 \le \frac{1}{(1-\omega )^2} \left( 1 + \tau \right) ^2 \Vert B\Vert _\textrm{F}^2. \end{aligned}$$

Combine the three bounds above to obtain the announced inequality. $\square$

We conclude this section with two bounds on the quantities $\delta$ and $\tau$, showing that they are close to zero in the regime (28).

#### Lemma 3.15

Suppose that $\lambda _d> 0$ and $\lambda _d> \lambda _{d+ 1}$. For all $n \in \{1, \dots , \ell \}$, the quantities $\delta$ and $\tau$, as defined in (29), are bounded as

$$\begin{aligned} \delta \le \big (1 + \varepsilon /\lambda _d\big )^{\ell - 1} - 1 \qquad {\text {and}} \qquad \tau \le \big (1 + \varepsilon /\lambda _d\big )^\ell - 1, \end{aligned}$$

where $$\varepsilon = \max _{j = d+ 1, \dots , {\tilde{d}}} \,|\lambda _j|$$.

#### Proof

Let  be the roots of the polynomial $\varsigma _n$. Then for all $x \le \lambda _d$ we have

$$\begin{aligned} |\varsigma _n(x) - 1| = \bigg | \prod _{i = 1}^n \big ( 1 - x/\gamma _i \big ) - 1 \bigg | \le \prod _{i = 1}^n \big ( 1 + |x|/\gamma _i \big ) - 1 \le \big ( 1 + |x|/\lambda _d\big )^n - 1. \end{aligned}$$

This gives the inequality for $\tau$. A similar argument yields the inequality for $\delta$. $\square$

## Application to trust-region algorithms

In this section we study the local convergence of trust-region algorithms (TR) to non-isolated minima, assuming the Polyak–Łojasiewicz condition (PŁ). We secure superlinear convergence when the subproblem solver is tCG (Algorithm 1). For this, we leverage the new analysis of CG developed in Sect. 3.

We let $\textrm{R}:\textrm{T}\mathcal {M}\rightarrow \mathcal {M}$ denote a retraction [3, Sect. 4.1], and use the notation $\textrm{R}_x(s) = \textrm{R}(x, s)$ for convenience. This is a smooth map from the tangent bundle $\textrm{T}\mathcal {M}$ such that $\textrm{R}_x(0) = x$ and $\textrm{D}\textrm{R}_x(0) = I$ for all $x \in \mathcal {M}$. In the Euclidean case we usually pick $\textrm{R}_x(s) = x + s$.

TR produces a sequence $\{(x_k, \Delta _k)\}$, where $x_k$ is the current iterate and $\Delta _k$ is the trust-region radius. At iteration *k*, we define a second-order model of $f$ around $x_k$ as

TRM $$\begin{aligned} m_k(s) = f(x_k) + \langle s, \nabla f(x_k)\rangle + \frac{1}{2}\langle s, H_k[s]\rangle , \end{aligned}$$

where $H_k$ is a linear map close to $\nabla ^2f(x_k)$—see Assumption A3. From this model, a step $s_k$ is computed by (usually approximately) solving the trust-region subproblem

TRS $$\begin{aligned} \min _{s_k \in \textrm{T}_{x_k}\mathcal {M}} m_k(s_k) \qquad {\text {subject to}} \qquad \Vert s_k\Vert \le \Delta _k. \end{aligned}$$

The point $x_k$ and radius $\Delta _k$ are then updated depending on the ratio

31 $$\begin{aligned} \rho _k = \frac{f(x_k) - f(\textrm{R}_{x_k}(s_k))}{m_k(0) - m_k(s_k)}. \end{aligned}$$

(If the denominator is zero, we let $\rho _k = 1$.) Specifically, given parameters  and $\bar{\Delta }> 0$, the update rules for the state are

32 $$\begin{aligned} x_{k + 1} = {\left\{ \begin{array}{ll} \textrm{R}_{x_k}(s_k) & {\text {if }}\, \rho _k> \rho ',\\ x_k & {\text {otherwise}}, \end{array}\right. } \qquad \Delta _{k + 1} = {\left\{ \begin{array}{ll} \frac{1}{4}\Delta _k & {\text {if }}\, \rho _k < \frac{1}{4},\\ \min (2\Delta _k, \bar{\Delta }) & {\text {if }}\, \rho _k > \frac{3}{4} \,{\text { and }}\, \Vert s_k\Vert = \Delta _k,\\ \Delta _k & {\text {otherwise}}. \end{array}\right. } \end{aligned}$$

An iteration *k* is *successful* when $\rho _k > \rho '$.

The main result of this section was stated as Theorem 1.2, with three assumptions around a local minimum $\bar{x}$. The first two require that the Hessian is sufficiently regular (note that A1 implies A2 with $L_H'= L_H$ when $\textrm{R}= \textrm{Exp}$—see for example [9, Cor. 10.56]). The last assumption further requires that the map $H_k$ is sufficiently close to $\nabla ^2f(x_k)$. It holds in particular if $H_k = \nabla ^2f(x_k)$ or $H_k = \nabla ^2(f\circ \textrm{R}_{x_k})(0)$.

### A1

The Hessian $\nabla ^2f$ is locally $L_H$-Lipschitz continuous around $\bar{x}$ for some $L_H\ge 0$.

### A2

There exists a constant $L_H'\ge 0$ such that the Lipschitz-type inequality

33 $$\begin{aligned} f(\textrm{R}_{x}(s)) - f(x) - \langle s, \nabla f(x)\rangle - \frac{1}{2}\langle s, \nabla ^2f(x)[s]\rangle \le \frac{L_H'}{6}\Vert s\Vert ^3 \end{aligned}$$

holds for all *x* sufficiently close to $\bar{x}$ and all *s* sufficiently small.

### A3

The linear maps $H_0, H_1, \ldots$ in (TRM) are symmetric. There is a constant $\beta _H\ge 0$ such that

34 $$\begin{aligned} \Vert H_k - \nabla ^2f(x_k)\Vert \le \beta _H\Vert \nabla f(x_k)\Vert \end{aligned}$$

whenever the iterate $x_k$ is sufficiently close to $\bar{x}$.

### The subproblem solver tCG satisfies C0, C1 and C2

Recall that tCG refers to Algorithm 1 and CG refers to the standard conjugate gradient algorithm, that is, tCG without the two truncation parts. Given an iterate $x_k$, we feed $A= H_k$, $b= -\nabla f(x_k)$ and $\Delta = \Delta _k$ to the subproblem solver tCG. It runs a certain number $n \le \dim \mathcal {M}$ of iterations and we set the step $s_k$ to be the resulting tCG iterate $v_n$.

In Sect. 1.1 we introduced the conditions C0, C1 and C2. Here we prove that tCG satisfies those three conditions under (PŁ). Given an iterate $x_k$, the Cauchy step is the minimizer of the subproblem (TRS) with the additional constraint that $s_k \in {{\,\textrm{span}\,}}\nabla f(x_k)$. It satisfies the sufficient decrease condition C0 with constant $c_0= 1/2$ [13, Thm. 6.3.1]. The Cauchy step happens to be the first iterate of tCG, and subsequent iterates can only improve. So, it follows that tCG also satisfies C0 with $c_0= 1/2$—see [13, Sect. 7.5.1]. The rest of this section focuses on conditions C1 and C2.

**Local behavior of CG.** To analyze tCG we rely on the developments from Sect. 3. Suppose that $f$ is $\mu$-(PŁ) around a local minimum $\bar{x}$. Then, since $f$ is ${\textrm{C}^{2}}$, it is also $\mu$-($\text {MB}$) at $\bar{x}$, as explained in Sect. 1.2. (Note that when $f$ is only ${\textrm{C}^{1}}$ its solution set may not be differentiable, even under PŁ.) In particular, the set $\mathcal {S}$ of local minima (1) is a smooth submanifold of $\mathcal {M}$ around $\bar{x}$ and $\dim \mathcal {S}= \dim \ker \nabla ^2f(\bar{x})$. Let $d$ be the codimension of $\mathcal {S}$ around $\bar{x}$. The $d$ nontrivial eigenvalues of $\nabla ^2f(\bar{x})$ are at least $\mu > 0$. The continuity of the eigenvalues of $\nabla ^2f$ implies that there exists a neighborhood $\mathcal {U}$ of $\bar{x}$ such that for all $x \in \mathcal {U}$ the orthogonal projector *P*(*x*) onto the top $d$ eigenspace of $\nabla ^2f(x)$ is well defined. We first recall that $\nabla f(x)$ aligns primarily with that eigenspace when *x* is close to $\bar{x}$.

#### Lemma 4.1

Suppose (PŁ) and A1 hold around $\bar{x}\in \mathcal {S}$. Then, with the same notation as above,

$$\begin{aligned} \nabla f(x)&= P(x) \nabla f(x) + O\big (\Vert \nabla f(x)\Vert ^2\big ) \end{aligned}$$

as $$x \rightarrow \bar{x}$$ and $$x \in \mathcal {U}$$.

#### Proof

See [43, Lem. 2.14]. $$\square $$

The algorithm tCG is CG with three stopping criteria. The first two (lines 10–13) trigger when the iteration is not well defined (that is, a non-positive eigenvalue is detected) or when the iterate exits the trust region. In these cases the output of tCG lies at the boundary of the trust region. The third stopping criterion (lines 16–18) triggers if the residual norm is small. In this case the output of tCG is the iterate of CG.

The conditions C1 and C2 bound the norms of the iterate and its residual for the output of tCG. To secure them we need the third stopping criterion to trigger (before the two others do), as otherwise we cannot control the norm of the output (which would lie at the boundary of the trust region). The purpose of the next lemma is to argue that this indeed happens around local minima where PŁ holds. To do this, we identify a particular iteration of CG with appropriate bounds on both the iterate and the residual. This is because CG implicitly filters out the small eigenvalues for a certain number of iterations. This result is central to establish the upcoming convergence guarantees of TR with tCG. From this, we argue later in this section that it is indeed the third stopping criterion that triggers.

#### Lemma 4.2

Assume that $f$ is $\mu$-(PŁ) and $\nabla ^2f$ is locally Lipschitz (A1) around $\bar{x}\in \mathcal {S}$. Let $d$ be the codimension of $\mathcal {S}$ around $\bar{x}$. Given $\mu ^\flat < \mu$,  and $\beta _H\ge 0$, there exists a neighborhood $\mathcal {U}$ of $\bar{x}$ such that for each $x \in \mathcal {U}$ and symmetric map $H :\textrm{T}_x\mathcal {M}\rightarrow \textrm{T}_x\mathcal {M}$ with $\Vert H - \nabla ^2f(x)\Vert \le \beta _H\Vert \nabla f(x)\Vert$ the following holds. There exists an integer $n \in \{0, \dots , d\}$ such that the *n*th iteration of CG on $(H, -\nabla f(x))$ is well defined and, moreover, the CG iterate $v_n$ and residual $r_n$ satisfy

35 $$\begin{aligned} \Vert v_n\Vert \le \frac{1}{\mu ^\flat }\Vert \nabla f(x)\Vert \qquad {\text {and}} \qquad \Vert r_n\Vert \le \Vert \nabla f(x)\Vert ^{1 + \theta }. \end{aligned}$$

#### Proof

Apply [43, Cor. 2.17] to confirm that $f$ satisfies $\mu$-($\text {MB}$) at $\bar{x}$. Let ${\tilde{d}}$ be the dimension of $\mathcal {M}$ (the domain of $f$) and let $d\le {\tilde{d}}$ be the codimension of the solution set $\mathcal {S}$ (around $\bar{x}$). For each $x \in \mathcal {M}$ we need to control the eigenvalues of all symmetric linear maps $H :\textrm{T}_x \mathcal {M}\rightarrow \textrm{T}_x \mathcal {M}$ which satisfy $\Vert H - \nabla ^2f(x)\Vert \le \beta _H\Vert \nabla f(x)\Vert$. To this end, we define three functions to control the eigenvalues of the map *H* depending on *x*. First, introduce

$$\begin{aligned} \lambda _-(x) = \lambda _d\big (\nabla ^2f(x)\big ) - \beta _H\Vert \nabla f(x)\Vert \qquad {\text {and}} \qquad \lambda _+(x) = \lambda _1\big (\nabla ^2f(x)\big ) + \beta _H\Vert \nabla f(x)\Vert \end{aligned}$$

to bracket the top $d$ eigenvalues of $H$. Then, let

$$\begin{aligned} \varepsilon (x) = \beta _H\Vert \nabla f(x)\Vert + \max _{j = d+ 1, \dots , {\tilde{d}}} \big |\lambda _j\big (\nabla ^2f(x)\big )\big | \end{aligned}$$

to bound the last ${\tilde{d}}- d$ eigenvalues. Standard theory for eigenvalue perturbation confirms that

$$\begin{aligned} \lambda _-(x) \le \lambda _d(H) \le \lambda _1(H) \le \lambda _+(x) \qquad {\text {and}} \qquad \max _{j = d+ 1, \dots , {\tilde{d}}} |\lambda _j(H)| \le \varepsilon (x). \end{aligned}$$

The functions $\lambda _-$, $\lambda _+$ and $\varepsilon$ are continuous. They also satisfy $\lambda _-(\bar{x}) \ge \mu$, $\lambda _+(\bar{x}) = \Vert \nabla ^2f(\bar{x})\Vert$ and $\varepsilon (\bar{x}) = 0$. There exists a neighborhood $\mathcal {U}$ of $\bar{x}$ such that $\varepsilon (x) < \lambda _-(x)$ for all $x \in \mathcal {U}$. In particular, for all $x \in \mathcal {U}$ and map *H* as prescribed, the orthogonal projector $P_H$ onto the top $d$ eigenspace of *H* is well defined. Let also *P*(*x*) denote the orthogonal projector onto the top $d$ eigenspace of $\nabla ^2f(x)$. Using the equality^3^$\Vert P_H - P(x)\Vert = \Vert P(x) (I - P_H)\Vert$, Davis–Kahan’s theorem [7, Thm. VII.3.1] implies that

$$\begin{aligned} \Vert P_H - P(x)\Vert \le \frac{\Vert H - \nabla ^2f(x)\Vert }{\lambda _d\big (\nabla ^2f(x)\big ) - \varepsilon (x)} \le \frac{\beta _H\Vert \nabla f(x)\Vert }{\lambda _d\big (\nabla ^2f(x)\big ) - \varepsilon (x)}. \end{aligned}$$

Combining this inequality with Lemma 4.1 yields that there exists a constant $C \ge 0$ such that

36 $$\begin{aligned} \Vert \nabla f(x) - P_H \nabla f(x)\Vert ^2 \le C \Vert \nabla f(x)\Vert ^4 \end{aligned}$$

for all *x* sufficiently close to $\bar{x}$ and map *H* as prescribed. If need be, restrict $\mathcal {U}$ to a smaller neighborhood of $\bar{x}$ so that (36), $\Vert \nabla f(x)\Vert ^2 \le 1$ and $C \Vert \nabla f(x)\Vert ^2 \le 1$ hold for all *x* in $\mathcal {U}$. We define the functions

37 $$\begin{aligned} u(x) = \Vert \nabla f(x)\Vert ^2\big (1 - C \Vert \nabla f(x)\Vert ^2\big ) \qquad {\text {and}} \qquad \bar{u}(x) = C \Vert \nabla f(x)\Vert ^4 \end{aligned}$$

on $\mathcal {U}$. From (36) we deduce that for all $x \in \mathcal {U}$ and map *H* as prescribed we have

38 $$\begin{aligned} \Vert (I - P_H) \nabla f(x)\Vert ^2 \le \bar{u}(x) \qquad {\text {and}} \qquad u(x) \le \Vert P_H \nabla f(x)\Vert ^2 \le \Vert \nabla f(x)\Vert ^2, \end{aligned}$$

where we used the identity $\Vert P_H \nabla f(x)\Vert ^2 = \Vert \nabla f(x)\Vert ^2 - \Vert (I - P_H) \nabla f(x)\Vert ^2$ for the lower-bound on the right. At a point *x* with appropriate map *H* we aim to invoke Lemma 3.14 with parameter $c(x, H) = \lambda _d(H)^{-1}u(x)^{\frac{3 + \theta }{2}}$. (Notice that $u(x) \le 1$, hence $c(x, H) \le \lambda _d(H)^{-1}u(x)$; together with $u(x) \le \Vert P_H \nabla f(x)\Vert ^2$, this will ensure that *c*(*x*, *H*) is an appropriate choice of *c* for Lemma 3.14.) To this end, consider the bounds in Lemma 3.15 and define

$$\begin{aligned} \delta (x)&= \big (1 + \varepsilon (x)/\lambda _-(x)\big )^{d- 1} - 1, \\ \eta (x)&= \tau (x) + \big (1 + \tau (x)\big ) \big (1 + \delta (x)\big )^2 \bar{u}(x)/u(x)^{\frac{3 + \theta }{2}},\\ \tau (x)&= \big (1 + \varepsilon (x)/\lambda _-(x)\big )^d- 1, \\ \omega (x)&= \big (1 + \eta (x)\big ) \big (1 + \delta (x)\big ) \bar{u}(x)/u(x)^{\frac{3 + \theta }{2}}. \end{aligned}$$

These functions are continuous around $\bar{x}$ and their value at $\bar{x}$ is zero since, by (37),

$$\begin{aligned} \bar{u}(x)/u(x)^{\frac{3 + \theta }{2}} = C \Vert \nabla f(x)\Vert ^{1 - \theta }\big (1 - C \Vert \nabla f(x)\Vert ^2\big )^{-(3 + \theta )/2} \end{aligned}$$

for all $x \in \mathcal {U}$. For the hypotheses of Lemma 3.14 to hold, we further restrict the neighborhood $\mathcal {U}$ so that $\omega (x) < 1$ for all $x \in \mathcal {U}$. Now pick a specific $x \in \mathcal {U}$ with map *H* as prescribed. If $\nabla f(x) = 0$ then the iteration $n = 0$ satisfies the requirements (35). Suppose now that $\nabla f(x) \ne 0$. Apply Lemma 3.14 to the pair $(H, -\nabla f(x))$ with $c = c(x, H)$. Combine it with the bounds given in Lemma 3.15. It yields the existence of an integer *n* such that *(i)* the *n*th iteration of CG is well defined, *(ii)* the iterate $v_n$ satisfies $\Vert v_n\Vert \le \lambda _-(x)^{-1}(1 - \omega (x))^{-1}\Vert \nabla f(x)\Vert$, and *(iii)* the residual $r_n$ satisfies

$$\begin{aligned} \Vert r_n\Vert ^2&\le \big (1 - \omega (x)\big )^{-2} \bigg (\frac{\lambda _+(x)}{\lambda _-(x)}u(x)^{\frac{3 + \theta }{2}} + \big (1 + \eta (x)\big )^2\big (1 + \delta (x)\big )^2 \bar{u}(x)^2 / u(x)^{\frac{3 + \theta }{2}}\\&\quad + \big (1 + \tau (x)\big )^2 \bar{u}(x) \bigg ). \end{aligned}$$

First notice that the bound $\Vert v_n\Vert \le \Vert \nabla f(x)\Vert /\mu ^\flat$ holds when *x* is sufficiently close to $\bar{x}$ because $\lambda _-(\bar{x}) \ge \mu > \mu ^\flat$ and $\omega (\bar{x}) = 0$. Now consider the residual norm. When *x* is sufficiently close to $\bar{x}$ the following inequalities hold:

$$\begin{aligned}&u(x)^{\frac{3 + \theta }{2}} \le \Vert \nabla f(x)\Vert ^{3 + \theta }, \bar{u}(x)^2 / u(x)^{\frac{3 + \theta }{2}} \le 2 C^2 \Vert \nabla f(x)\Vert ^{5 - \theta } \\&{\text {and}}\,\, \bar{u}(x) \le C \Vert \nabla f(x)\Vert ^4. \end{aligned}$$

We deduce that $\Vert r_n\Vert \le \Vert \nabla f(x)\Vert ^{1 + \theta }$ if *x* is sufficiently close to $\bar{x}$ because $3 + \theta > 2 + 2 \theta$. (The strict inequality $\theta < 1$ plays a role here, making it possible to absorb multiplicative factors.) $\square$

Note that the conclusion of Lemma 4.2 is immediate if one makes the stronger assumption $\nabla ^2f(\bar{x}) \succ 0$. In that case we can choose the iteration *n* to be the grade of $(H, -\nabla f(x))$, as per Definition 2.1, to obtain the requirements on $v_n$ and $r_n$. However, this is in general not possible when only PŁ holds. This is because after a certain number of iterations, CG starts to detect the small eigenvalues of $H$. When this happens, the iterates of the algorithm typically explode and the bounds in (35) no longer hold. We illustrate this phenomenon in Figures 2 and 3. Remarkably, tCG stops automatically before that happens, as we argue subsequently.

#### Remark 4.3

Lemma 4.2 does not specify the size of the neighborhood $\mathcal {U}$ in which the conclusion holds because we have only little control over it. Notice that the magnitudes of $\delta$ and $\tau$, as defined in (29), might depend on the dimension of the problem, as reflected by Lemma 3.15. This induces for the size of $\mathcal {U}$ a dependency on the codimension of $\mathcal {S}$. However, the poor bounds for $\delta$ and $\tau$ in Lemma 3.15 are due to the *negative* eigenvalues. As a result, it should be possible to derive much sharper bounds in the regions near $\bar{x}$ where all the eigenvalues are positive.

**Application to TR with tCG.** A first consequence of Lemma 4.2 is that TR with tCG satisfies C1 around points where PŁ holds. To show this, we use the following classical result.

#### Lemma 4.4

The iterates of CG and tCG grow in norm: $0 = \Vert v_0\Vert \le \Vert v_1\Vert \le \Vert v_2\Vert \le \cdots$.

#### Proof

See [13, Thm. 7.5.1] or [38, Thm. 7.3]. $\square$

#### Proposition 4.5

Consider tCG with parameters $\kappa > 0$ and . Assume that A1, A3 and $\mu$-(PŁ) hold around $\bar{x}\in \mathcal {S}$. Given $\mu ^\flat < \mu$, there exists a neighborhood of $\bar{x}$ where the tCG steps satisfy C1 with constant $c_1= 1/\mu ^\flat$.

#### Proof

Let $\mathcal {U}$ be a neighborhood of $\bar{x}$ as in Lemma 4.2 for the parameters $\mu ^\flat$, $\theta$ and $\beta _H$ (A3). Shrink $\mathcal {U}$ so that $\Vert \nabla f(x)\Vert ^\theta \le \kappa$ for all $x \in \mathcal {U}$. Given an iterate $x_k \in \mathcal {U}$ with $H_k$, let *n* be as provided by Lemma 4.2 (so the bounds (35) hold). Algorithm 1 on $(H_k, -\nabla f(x_k))$ terminates either with $v_n$ (because the residual norm stopping criterion on line 16 triggers), or earlier. In all cases, Lemma 4.4 implies the returned tCG step has norm at most that of $v_n$, which itself is at most $\frac{1}{\mu ^\flat }\Vert \nabla f(x_k)\Vert$. $\square$

We now leverage Lemma 4.2 to establish C2. The residual of CG satisfies $r_n = -\nabla m(v_n)$, where $m$ is the model (TRM). It follows that (35) also provides a bound on $\nabla m(v_n)$. We first state a helper lemma.

#### Lemma 4.6

Suppose that A2 and A3 hold around $\bar{x}\in \mathcal {S}$. Also assume that the steps $s_k$ satisfy C0 and C1 around $\bar{x}$. For all $\varepsilon > 0$ there exists a neighborhood $\mathcal {U}$ of $\bar{x}$ such that if an iterate $x_k$ is in $\mathcal {U}$ then $\rho _k \ge 1 - \varepsilon$, where $\rho _k$ is the ratio (31).

#### Proof

See [43, Prop. 4.10]. $$\square $$

#### Proposition 4.7

Let $\{x_k\}$ be a sequence that TR generates using the tCG subproblem solver (Algorithm 1) with parameters $\kappa > 0$ and . Suppose that $\{x_k\}$ converges to a point $\bar{x}\in \mathcal {S}$ around which $f$ is $\mu$-(PŁ). Also assume that A1, 33 and A3 hold around $\bar{x}$. Then the sequence $\{(x_k, s_k)\}$ satisfies C2 with constants $c_2= 1$ and $\theta$.

#### Proof

The subproblem solver tCG satisfies C0 and C1 around $\bar{x}$ (see the beginning of Sect. 4.1 and Proposition 4.5). Lemma 4.6 gives that $\liminf _{k \rightarrow +\infty } \rho _k \ge 1$. This implies that the radii $\{\Delta _k\}$ are bounded away from zero because the update mechanism (32) does not decrease the radius when $\rho _k \ge \frac{1}{4}$. Given $\mu ^\flat < \mu$, the tCG parameter $\theta$, and the constant $\beta _H$ from A3, let $\mathcal {U}$ be a neighborhood of $\bar{x}$ as in Lemma 4.2. There exists an integer *K* such that for all $k \ge K$ we have

$$\begin{aligned} x_k \in \mathcal {U}, \qquad \Vert \nabla f(x_k)\Vert ^\theta \le \kappa \qquad {\text {and}} \qquad \frac{1}{\mu ^\flat }\Vert \nabla f(x_k)\Vert < \Delta _k. \end{aligned}$$

Now let $k \ge K$. Lemma 4.2 provides an integer *n* such that the *n*th iteration of CG on $(H_k, -\nabla f(x_k))$ is well defined. Moreover, the *n*th iterate $v_n$ and residual $r_n$ satisfy

$$\begin{aligned} \Vert v_n\Vert \le \frac{1}{\mu ^\flat }\Vert \nabla f(x_k)\Vert < \Delta _k \qquad {\text {and}} \qquad \Vert r_n\Vert \le \Vert \nabla f(x_k)\Vert ^{1 + \theta }. \end{aligned}$$

The residual norm $\Vert r_n\Vert$ is compatible with the termination criterion in line 16. We deduce that tCG performs a certain number $p \le n$ of iterations on the inputs $(H_k, -\nabla f(x_k))$. Recall that the iterates grow in norm (Lemma 4.4). Hence at iteration *p* the termination criteria in line 10 are incompatible with *(i)* the fact that the *p*th iteration is well defined and *(ii)* the inequality $\Vert v_p\Vert \le \Vert v_n\Vert < \Delta _k$. It follows that tCG must terminate from the stopping criterion on the residual norm in line 16. We obtain the inequality $\Vert \nabla m_k(s_k)\Vert \le \Vert \nabla f(x_k)\Vert ^{1 + \theta }$, and so C2 holds. $\square$

### Capture and order of convergence

In this section we consider a general subproblem solver compatible with C0, C1 and C2, with tCG as the motivating example. We derive a capture result and superlinear rates of convergence.

**Capture of the iterates.** First, we show that C0 and C1 imply that the iterates of TR locally satisfy a condition known as the *strong decrease property* (41) (see [1] and also [43, Sect. 3.2] for more literature). The proofs below combine the bound C1 and arguments that appear in [1, Thm. 4.4].

#### Lemma 4.8

Suppose that A3 holds around $\bar{x}\in \mathcal {S}$. Given $\lambda ^\sharp > \lambda _{\max }(\nabla ^2f(\bar{x}))$, there exists a neighborhood $\mathcal {U}$ of $\bar{x}$ such that if an iterate $x_k$ is in $\mathcal {U}$ then

39 $$\begin{aligned} \frac{\Vert \nabla f(x_k)\Vert ^3}{\langle \nabla f(x_k), H_k[\nabla f(x_k)]\rangle } \ge \frac{1}{\lambda ^\sharp }\Vert \nabla f(x_k)\Vert . \end{aligned}$$

(We define the expression on the left as $+\infty$ when the denominator is zero.)

#### Proof

Let $\mathcal {U}$ be a neighborhood of $\bar{x}$ such that $\Vert \nabla ^2f(x)\Vert + \beta _H\Vert \nabla f(x)\Vert \le \lambda ^\sharp$ for all $x \in \mathcal {U}$, where $\beta _H$ is the constant that appears in A3. Suppose that $x_k$ is in $\mathcal {U}$. Cauchy–Schwarz yields

$$\begin{aligned} \langle \nabla f(x_k), H_k[\nabla f(x_k)]\rangle \le \lambda ^\sharp \Vert \nabla f(x_k)\Vert ^2 \end{aligned}$$

because $\Vert H_k\Vert \le \Vert \nabla ^2f(x_k)\Vert + \Vert H_k - \nabla ^2f(x_k)\Vert \le \lambda ^\sharp$. We obtain (39) from this. $\square$

#### Lemma 4.9

Suppose C1 holds in a neighborhood $\mathcal {U}$ of $\bar{x}\in \mathcal {S}$. Possibly after restricting $\mathcal {U}$, there exists $c_r\ge 1$ such that

40 $$\begin{aligned} {{\,\textrm{dist}\,}}(\textrm{R}_{x_k}(s_k), x_k) \le c_r\Vert s_k\Vert \end{aligned}$$

for all iterates $x_k$ in $\mathcal {U}$ and steps $s_k$, where ${{\,\textrm{dist}\,}}$ is the Riemannian distance on $\mathcal {M}$.

#### Proof

This is a consequence of [44, Lem. 6] because $\textrm{R}$ is smooth. $\square$

In the Euclidean case, ${{\,\textrm{dist}\,}}(x, y) = \Vert x - y\Vert$ and for $\textrm{R}_x(s) = x+s$ we can clearly take $c_r= 1$. In general, we can take $c_r> 1$ as close to 1 as desired after sufficiently restricting the neighborhood.

#### Lemma 4.10

Assume that A3 holds around a local minimum $\bar{x}\in \mathcal {S}$. Let TR generate iterates $\{x_k\}$ using a subproblem solver satisfying C0 and C1 with constants $c_0$ and $c_1$ around $\bar{x}$. Given $\lambda ^\sharp > \lambda _{\max }(\nabla ^2f(\bar{x}))$, there exists a neighborhood $\mathcal {U}$ of $\bar{x}$ such that if $x_k$ is in $\mathcal {U}$ then

41 $$\begin{aligned} \begin{aligned}&f(x_k) - f(x_{k + 1}) \ge \frac{c_0\rho '}{c_r} \min \!\Big (1, \frac{1}{c_1\lambda ^\sharp }\Big ) \Vert \nabla f(x_k)\Vert {{\,\textrm{dist}\,}}(x_k, x_{k + 1})\\&{\text {and}}\quad x_k \in \mathcal {S}\;\;\Rightarrow \;\; x_{k + 1} = x_k, \end{aligned} \end{aligned}$$

where $\rho '$ is as in the algorithm definition (32) and $c_r$ is as in Lemma 4.9.

#### Proof

The implication $x_k \in \mathcal {S}\Rightarrow x_{k + 1} = x_k$ is immediate because the gradient is zero on the optimal set $\mathcal {S}$ and C1 then implies that $s_k = 0$. We now prove the lower-bound on the function decrease. Let $\mathcal {U}$ be a neighborhood of $\bar{x}$ where the inequalities in C1, (39) and (40) hold. Consider $x_k \in \mathcal {U}$. Starting from the inequality in C0, apply (39) then C1 to confirm that

42 $$\begin{aligned} m_k(0) - m_k(s_k) \ge c_0\min \!\left( \Delta _k, \frac{\Vert s_k\Vert }{c_1\lambda ^\sharp }\right) \Vert \nabla f(x_k)\Vert . \end{aligned}$$

Since $\Delta _k \ge \Vert s_k\Vert$, factor $\Vert s_k\Vert$ out of the min and use ${{\,\textrm{dist}\,}}(x_k, x_{k + 1}) \le c_r\Vert s_k\Vert$ (owing to Lemma 4.9) to claim

43 $$\begin{aligned} m_k(0) - m_k(s_k) \ge \frac{c_0}{c_r} \min \!\left( 1, \frac{1}{c_1\lambda ^\sharp }\right) \Vert \nabla f(x_k)\Vert {{\,\textrm{dist}\,}}(x_k, x_{k+1}). \end{aligned}$$

Now two cases can happen from the TR dynamics (32). Either the step is rejected ($x_{k+1} = x_k$) and (41) holds trivially. Or the step is accepted ($x_{k+1} = \textrm{R}_{x_k}(s_k)$) because $f(x_k) - f(\textrm{R}_{x_k}(s_k)) \ge \rho '\big (m_k(0) - m_k(s_k)\big )$, in which case (41) also holds. (Note: $\rho '$ could be improved to $1-o(1)$.) $\square$

Assuming additionally that $f$ is $\mu$-(PŁ) and $\nabla ^2f$ is locally Lipschitz around $\bar{x}$ (A1), and given $\mu ^\flat < \mu$, we know that tCG satisfies C1 with constant $1/\mu ^\flat$ (Proposition 4.5). In this case we can specialize Lemma 4.10 and obtain (41) with constants $c_0= 1/2$ and $c_1= 1/\mu ^\flat$.

The strong decrease property notably leads to a capture result: see [1] and comments in [43, Sect. 3.2]. To secure it, we need to ensure that TR accumulates only at critical points. That is effectively the case. However, to formalize this globally we would need a few technical assumptions (see for example [3, Thm. 7.4.4] and [9, Prop. 6.25]). Fortunately, we only need this property locally around local minima with a PŁ condition. As it turns out, this frees us from those technicalities.

#### Proposition 4.11

(Capture) Suppose that A2, A3 and (PŁ) hold around $\bar{x}\in \mathcal {S}$. Let TR generate a sequence $\{x_k\}$ with a subproblem solver that satisfies C0 and C1. Given a neighborhood $\mathcal {U}$ of $\bar{x}$, there exists a neighborhood $\mathcal {V} \subseteq \mathcal {U}$ of $\bar{x}$ such that if an iterate of TR enters $\mathcal {V}$ then all the subsequent iterates stay in $\mathcal {U}$ and the sequence converges to some point $x_\infty \in \mathcal {U} \cap \mathcal {S}$.

#### Proof

We can restrict $\mathcal {U}$ so that all critical points in $\mathcal {U}$ are in $\mathcal {S}$ because (PŁ) holds around $\bar{x}$. We want to apply [43, Cor. 3.7] to $\mathcal {U}$. This requires the algorithm to satisfy two properties called *vanishing steps* and *bounded path length* in that reference. The vanishing steps property holds around $\bar{x}$ because we assume C1. Moreover, the strong decrease from Lemma 4.10 together with (PŁ) imply the bounded path length property (see [43, Lem 3.8]). To apply the stated corollary, we finally need to show that if $\{x_k\}$ accumulates at a point $x_\infty \in \mathcal {U}$ then $x_\infty \in \mathcal {S}$. Restrict $\mathcal {U}$ so that if an iterate $x_k$ is in $\mathcal {U}$ then the associated ratio satisfies $\rho _k \ge \frac{1}{4}$ (Lemma 4.6). Given a number $\lambda ^\sharp > \lambda _{\max }(\nabla ^2f(\bar{x}))$, further restrict $\mathcal {U}$ so that the conclusions of Lemmas 4.8 and 4.10 hold. Now invoke [43, Prop. 3.5]. We obtain an open neighborhood $\mathcal {V} \subseteq \mathcal {U}$ of $\bar{x}$ such that if an iterate enters $\mathcal {V}$ then all subsequent iterates stay in $\mathcal {U}$. Suppose that the sequence $\{x_k\}$ accumulates at some point $x_\infty \in \mathcal {V}$. There exists an integer *K* such that $x_k \in \mathcal {U}$ for all $k \ge K$. As a result the trust-region radii eventually stop decreasing: there exists a number $\Delta _{\min } > 0$ such that $\Delta _k \ge \Delta _{\min }$ for *all* *k*. Suppose for contradiction that $\nabla f(x_\infty ) \ne 0$. Then, consider condition C0 together with (39), $\Delta _k \ge \Delta _{\min }$ and the fact that $\Vert \nabla f(x_k)\Vert$ is bounded away from zero along a subsequence convergent to $x_\infty$. From this, deduce the existence of a number $\omega > 0$ such that

$$\begin{aligned} f(x_k) - f(x_{k + 1}) = \rho _k \big (m_k(0) - m_k(s_k)\big ) \ge \omega \end{aligned}$$

for infinitely many iterations *k*, all successful owing to $\rho _k \ge 1/4$. This is incompatible with the facts that $\{x_k\}$ accumulates at $x_\infty$ and that $f(x_k)$ is decreasing. It follows that $\nabla f(x_\infty ) = 0$ and so $x_\infty \in \mathcal {S}$. $\square$

**Order of convergence.** Now that local convergence to a point is secured, we turn our focus to the convergence rate of TR. As we show, the conditions C0, C1 and C2 are sufficient to secure superlinear rates.

#### Proposition 4.12

Suppose that A1, A2 and A3 hold around $\bar{x}\in \mathcal {S}$. Let TR generate iterates $\{x_k\}$ converging to $\bar{x}$ using a subproblem solver satisfying C0, C1 and C2 around $\bar{x}$. Then the sequence $\{\Vert \nabla f(x_k)\Vert \}$ converges superlinearly to zero with order at least $1 + \theta$.

#### Proof

Let $c_0$, $c_1$ and $c_2$ be the constants associated to C0, C1 and C2. From Lemma 4.6 and (32) we deduce that all the steps are eventually successful. We consider large enough iterations *k* so that it is the case. Since ${{\,\textrm{dist}\,}}(x_k, x_{k+1}) \rightarrow 0$, the vector $\textrm{Log}_{x_k}(x_{k + 1})$ is well defined for all *k* large enough. (Recall from Table 1 that $\textrm{Log}_x(y) = y - x$ in the Euclidean case.) Let $v_k = s_k - \textrm{Log}_{x_k}(x_{k + 1})$ (this is zero in the Euclidean case). Let $\Gamma _{x_{k + 1}}^{x_k}$ denote parallel transport along the minimizing geodesic connecting $x_{k+1}$ to $x_{k}$—this too is well defined for large enough *k*. (In the Euclidean case, $\Gamma _{x_{k + 1}}^{x_k}$ is identity.) The triangle inequality then provides:

44 $$\begin{aligned} \Vert \nabla f(x_{k + 1})\Vert&= \big \Vert \Gamma _{x_{k + 1}}^{x_k}\nabla f(x_{k + 1}) - \nabla f(x_k) - \nabla ^2f(x_k)[\textrm{Log}_{x_k}(x_{k + 1})]\nonumber \\&\qquad - \nabla ^2f(x_k)[v_k] - \big (H_k - \nabla ^2f(x_k)\big )[s_k] + \nabla m_k(s_k)\big \Vert \nonumber \\&\le \frac{L_H}{2}{{\,\textrm{dist}\,}}(x_k, x_{k + 1})^2 + \Vert \nabla ^2f(x_k)\Vert \Vert v_k\Vert + \beta _H\Vert \nabla f(x_k)\Vert \Vert s_k\Vert \nonumber \\&\qquad + c_2\Vert \nabla f(x_k)\Vert ^{1 + \theta }, \end{aligned}$$

where we invoked the Lipschitz continuity of the Hessian A1 [9, Cor. 10.56], the bound in A3, and the bound in C2. The condition C1 together with Lemma 4.9 give that ${{\,\textrm{dist}\,}}(x_k, x_{k + 1}) \le c_r\Vert s_k\Vert \le c_rc_1\Vert \nabla f(x_k)\Vert$ for large enough *k*. We now bound the quantity $\Vert v_k\Vert$. There exists a neighborhood of the zero section of the tangent bundle such that $(x, s) \mapsto (x, \textrm{R}_x(s))$ is a diffeomorphism [9, Cor. 10.27]. Moreover, the inverse function theorem implies that $\textrm{D}\textrm{R}_x^{-1}(x) = I$ for all $x \in \mathcal {M}$ because $\textrm{D}\textrm{R}_x(0) = I$. It follows that there exists a neighborhood $\mathcal {U}$ of $\bar{x}$ such that for all $x, y \in \mathcal {U}$ we have

$$\begin{aligned} \textrm{R}_x^{-1}(y)&= \textrm{R}_x^{-1}(x) + \textrm{D}\textrm{R}_x^{-1}(x)[\textrm{Log}_x(y)] + O({{\,\textrm{dist}\,}}(x, y)^2) \\&= \textrm{Log}_x(y) + O({{\,\textrm{dist}\,}}(x, y)^2). \end{aligned}$$

In particular, using the identity $s_k = \textrm{R}_{x_k}^{-1}(x_{k + 1})$, we find that there exists a constant $c_v$ such that $\Vert v_k\Vert \le c_v{{\,\textrm{dist}\,}}(x_k, x_{k + 1})^2$ holds for large enough *k*. It follows from (44) that

$$\begin{aligned}&\Vert \nabla f(x_{k + 1})\Vert \le \bigg ( c_r^2 c_1^2 \Big (\frac{L_H}{2} + c_v \Vert \nabla ^2f(x_k)\Vert \Big ) + c_1\beta _H\bigg ) \Vert \nabla f(x_k)\Vert ^2 \\&+ c_2\Vert \nabla f(x_k)\Vert ^{1 + \theta } \end{aligned}$$

for large enough *k*, showing the superlinear convergence of the sequence $\{\Vert \nabla f(x_k)\Vert \}$. $\square$

From this result, we can deduce the superlinear convergence of the sequences $\{{{\,\textrm{dist}\,}}(x_k, \mathcal {S})\}$ and $\{f(x_k) - f(\bar{x})\}$ as follows. Suppose that (PŁ) holds around $\bar{x}$. This is equivalent to the error bound condition, meaning that $\mu {{\,\textrm{dist}\,}}(x, \mathcal {S}) \le \Vert \nabla f(x)\Vert$ for all *x* sufficiently close to $\bar{x}$ (see for example [25, Thm. 2] or [43, Rmk. 2.10]). This implies that $\{{{\,\textrm{dist}\,}}(x_k, \mathcal {S})\}$ converges to zero superlinearly with order at least $1 + \theta$. Finally we can deduce that $\{f(x_k) - f(\bar{x})\}$ converges to zero with the same rate from (PŁ).

#### Proof (Proof of Theorem 1.2)

First invoke Propositions 4.5 and 4.7 to obtain that tCG satisfies C1 and C2. The result then follows from Propositions 4.11 and 4.12. $\square$

#### Remark 4.13

Theorem 1.2 ensures superlinear convergence but not quadratic convergence. And indeed, without further assumptions on $f$ our proof cannot provide quadratic convergence for TR with tCG. The reason is that the parameter $\theta$ in Lemma 4.2 has to be *strictly* less than 1. We show here that this cannot be improved. Consider the function $f(x, y) = \frac{3}{16}(1 + \frac{64}{3}x^2)y^2$ and the path $c(\varepsilon ) = (\frac{1}{8}\sqrt{1 - \varepsilon }, \sqrt{\varepsilon })$ for a small parameter $\varepsilon \ge 0$. The function $f$ is polynomial and satisfies (PŁ) around *c*(0). Moreover, the eigenvalues of $\nabla ^2f(c(\varepsilon ))$ are both positive for $\varepsilon$ sufficiently small. Consider CG with inputs $\big (\nabla ^2f(c(\varepsilon )), -\nabla f(c(\varepsilon ))\big )$. Let $r_1(\varepsilon )$ denote the residual of the first iteration and $v_2(\varepsilon )$ the iterate of the second iteration. Then, together with the gradient, they satisfy

$$\begin{aligned} \Vert r_1(\varepsilon )\Vert \sim \varepsilon \qquad \Vert \nabla f(c(\varepsilon ))\Vert ^2 \sim \varepsilon /4 \qquad {\text {and}} \qquad \Vert v_2(\varepsilon )\Vert \sim 1/6\varepsilon \end{aligned}$$

as $\varepsilon \rightarrow 0$, where $\sim$ denotes asymptotic equivalence. In particular, the inequality $\Vert r_1(\varepsilon )\Vert \le \Vert \nabla f(c(\varepsilon ))\Vert ^2$ cannot hold when $\varepsilon$ is sufficiently close to zero, and $\Vert v_2(\varepsilon )\Vert / \Vert \nabla f(c(\varepsilon ))\Vert \rightarrow +\infty$ as $\varepsilon \rightarrow 0$. We conclude that both iterates of CG are incompatible with the bounds (35), even when $\varepsilon$ is arbitrarily close to zero. Since $\Vert v_2(\varepsilon )\Vert \rightarrow +\infty$ as $\varepsilon \rightarrow 0$, this also shows that tCG with parameter $\theta = 1$ suffers from the same shortcomings as the exact subproblem solver: it is not possible to ensure capture of the iterates (the condition C1 breaks).

## A note about quotient manifolds

Symmetry is a common source of non-isolated minima in optimization. Explicitly, suppose we seek to minimize $f:\mathcal {M}\rightarrow {\mathbb R}$ and there exists an equivalence relation $\sim$ on $\mathcal {M}$ such that $x \sim y \implies f(x) = f(y)$. Under certain (well-understood) circumstances, the quotient space $\mathcal {M}/{\sim }$ is itself a manifold, and all equivalence classes are submanifolds of $\mathcal {M}$ with equal dimension [3, Sect. 3.4.1].

In this case, any local minimizer $\bar{x}$ of $f$ belongs to an equivalence class $[\bar{x}] = \{ x \in \mathcal {M}: x \sim \bar{x}\}$ of points which are all local minimizers. If the symmetry stems from the action of a Lie group with positive dimension (e.g., rotations or translations), then each equivalence class is itself a submanifold of $\mathcal {M}$ of positive dimension.

Under those circumstances, the Hessian $\nabla ^2f(\bar{x})$ cannot be positive definite. However, the Hessian may well be positive definite upon passing to the quotient. Explicitly, let $\varphi :\mathcal {M}\rightarrow \mathcal {M}/{\sim }$ be the canonical projection $\varphi (x) = [x]$. Then, the symmetries of $f$ ensure that there exists a function $g:\mathcal {M}/{\sim } \rightarrow {\mathbb R}$ such that $f= g\circ \varphi$. If the symmetries are the only reason why $\nabla ^2f(\bar{x}) \not \succ 0$, then $\nabla ^2g([\bar{x}])$ is positive definite [9, Ex. 9.46]. This means that if we run optimization algorithms on the quotient manifold, then we can expect all the good convergence properties that normally come with a positive definite Hessian, including quadratic local convergence for TR-tCG. This forms part of the motivation for studying optimization on quotient manifolds—see [3] and [9, Ch. 9].

Under those same circumstances though, it is typical to observe that TR-tCG enjoys fast local convergence even if we disregard the symmetries and run the algorithm on $f:\mathcal {M}\rightarrow {\mathbb R}$ directly. We can now understand this as follows. If $\nabla ^2g([\bar{x}]) \succ 0$, then $f$ satisfies (PŁ) around $\bar{x}$ (see for example [43, Sect. 1.2]). Thus, Theorem 1.2 applies, guaranteeing superlinear local convergence for TR-tCG. The role of tCG appears to be instrumental.

To go beyond the assumption $\nabla ^2g([\bar{x}]) \succ 0$, note that when $\mathcal {M}/{\sim }$ is a *Riemannian* quotient [3, Sect. 3.6.2] of $\mathcal {M}$, the function $f$ is $\mu$-(PŁ) around a minimum $\bar{x}$ if and only if $g$ is $\mu$-(PŁ) around $[\bar{x}]$. This equivalence readily follows from the equalities $f(x) = g([x])$ and $\Vert \nabla f(x)\Vert = \Vert \nabla g([x])\Vert$ for all $x \in \mathcal {M}$. If $\mathcal {M}/{\sim }$ is a quotient manifold with another Riemannian metric, then the equivalence still holds but possibly with different PŁ constants.

## Figures and Tables

**Fig. 5:**
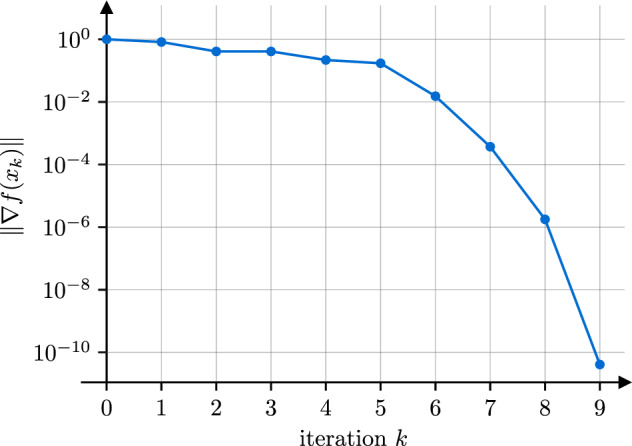
The gradient norm exhibits a pattern typical of superlinear convergence

## Simple example with superlinear convergence

Consider the cost function $f:{\mathbb R}^n \times {\mathbb R}^n \rightarrow {\mathbb R}$ defined as

$$\begin{aligned} f(x, y) = \frac{1}{2} \sum _{i = 1}^n \big (y_i - \sin (x_i)\big )^2 = \frac{1}{2}\Vert y - \sin (x)\Vert ^2, \end{aligned}$$

where $\sin$ is applied entrywise. The set of minimizers $\mathcal {S}= \big \{(x, y) \in {\mathbb R}^n \times {\mathbb R}^n : y = \sin (x)\big \}$ is an embedded submanifold of ${\mathbb R}^n \times {\mathbb R}^n$. Standard computations yield the gradient

$$\begin{aligned} \nabla f(x, y) = \Big (-\cos (x) \odot \big (y - \sin (x)\big ), y - \sin (x)\Big ). \end{aligned}$$

It is clear that $\Vert \nabla f(x, y)\Vert ^2 \ge \Vert y - \sin (x)\Vert ^2 = 2f(x, y)$, hence $f$ is globally 1-(PŁ).

We run TR-tCG with $n = 100$ and a random initialization. Figure 5 displays the norm of the gradient at the iterates. The convergence appears superlinear.

We now briefly argue that the Hessian has a negative eigenvalue in the vicinity of $\mathcal {S}$. Consider the case $n = 1$ for simplicity. The Hessian is the $2 \times 2$ matrix

$$\begin{aligned} \nabla ^2f(x, y) = \begin{bmatrix} \sin (x)\big (y - \sin (x)\big ) + \cos (x)^2 &  -\cos (x)\\ -\cos (x) &  1 \end{bmatrix}. \end{aligned}$$

There is a negative eigenvalue if the determinant $\sin (x)\big (y - \sin (x)\big )$ is negative. Given $(\bar{x}, \bar{y}) \in \mathcal {S}$, we can find points (*x*, *y*) arbitrarily close to $(\bar{x}, \bar{y})$ such that this quantity is negative.

## Footnotes

1 The contributions are relevant for $\mathcal {M}= \mathbb {R}^n$ too. We treat the more general manifold case as it involves only mild overhead in notation, summarized in Table 1.

2 It is easy to reason that $\zeta _n$ is the translation of a polynomial that naturally appears when studying the MINRES algorithm on the problem $(A- \lambda I, b)$.

3 This is because $\Vert P(x)(I - P_H)v\Vert \le \Vert (P_H - P(x))v\Vert$ for all *v*, with equality when *v* is an eigenvector of $P_H - P(x)$.

## References

[1] Absil, P.-A., Mahony, Robert, Andrews, Benjamin: Convergence of the iterates of descent methods for analytic cost functions. SIAM J. Optim. **16**(2), 531–547 (2005)

[2] Absil, P.-A., Baker, Christopher G., Gallivan, Kyle A.: Trust-region methods on Riemannian manifolds. Found. Comput. Math. **7**(3), 303–330 (2007)

[3] Absil, P.-A.: Robert Mahony, and Rodolphe Sepulchre. Princeton University Press, Optimization algorithms on matrix manifolds (2008)

[4] Adachi, Satoru, Iwata, Satoru, Nakatsukasa, Yuji, Takeda, Akiko: Solving the trust-region subproblem by a generalized eigenvalue problem. SIAM J. Optim. **27**(1), 269–291 (2017)

[5] Attouch, Hédy., Bolte, Jérôme., Redont, Patrick, Soubeyran, Antoine: Proximal alternating minimization and projection methods for nonconvex problems: an approach based on the Kurdyka–Łojasiewicz inequality. Math. Oper. Res. **35**(2), 438–457 (2010)

[6] Attouch, Hédy., Bolte, Jérôme., Svaiter, Benar Fux: Convergence of descent methods for semi-algebraic and tame problems: proximal algorithms, forward-backward splitting, and regularized Gauss–Seidel methods. Math. Program. **137**(1), 91–129 (2013)

[7] Bhatia, R.: Matrix Analysis. Springer, New York (1997). 10.1007/978-1-4612-0653-8

[8] Bolte, Jérôme., Sabach, Shoham, Teboulle, Marc: Proximal alternating linearized minimization for nonconvex and nonsmooth problems. Math. Program. **146**(1), 459–494 (2014)

[9] Boumal, Nicolas: An Introduction to Optimization on Smooth Manifolds. Cambridge University Press (2023)

[10] Carmon, Yair, Duchi, John C.: First-order methods for nonconvex quadratic minimization. SIAM Rev. **62**(2), 395–436 (2020)

[11] Cartis, Coralia, Gould, Nicholas IM., Toint, Philippe L.: Adaptive cubic regularisation methods for unconstrained optimization. Part I: motivation, convergence and numerical results. Math. Program. **127**(2), 245–295 (2011)

[12] Cartis, Coralia, Gould, Nicholas IM., Toint, Philippe L.: Adaptive cubic regularisation methods for unconstrained optimization. Part II: worst-case function- and derivative-evaluation complexity. Math. Program. **130**(2), 295–319 (2011)

[13] Conn, Andrew R, Gould, Nicholas IM, Toint, Philippe L: Trust Region Methods. SIAM, (2000)

[14] Dembo, Ron S., Steihaug, Trond: Truncated-Newton algorithms for large-scale unconstrained optimization. Math. Program. **26**(2), 190–212 (1983)

[15] Dembo, Ron S., Eisenstat, Stanley C., Steihaug, Trond: Inexact Newton methods. SIAM J. Numer. Anal. **19**(2), 400–408 (1982)

[16] Fan, Jinyan: Convergence rate of the trust region method for nonlinear equations under local error bound condition. Comput. Optim. Appl. **34**(2), 215–227 (2006)

[17] Fong, David Chin-Lung., Saunders, Michael: CG versus MINRES: an empirical comparison. Sultan Qaboos Univ. J. Sci. [SQUJS] **17**(1), 44–62 (2012)

[18] Golub, Gene H., Meurant, Gérard.: Matrices Moments and Quadrature with Applications. Princeton University Press (2010)

[19] Gould, Nicholas IM., Lucidi, Stefano, Roma, Massimo, Toint, Philippe L.: Solving the trust-region subproblem using the Lanczos method. SIAM J. Optim. **9**(2), 504–525 (1999)

[20] Greenbaum, Anne: Behavior of slightly perturbed Lanczos and conjugate-gradient recurrences. Linear Algebra Appl. **113**, 7–63 (1989)

[21] Greenbaum, A.: Iterative Methods for Solving Linear Systems. Society for Industrial and Applied Mathematics, (1997)

[22] Greenbaum, Anne, Strakos, Zdenek: Predicting the behavior of finite precision Lanczos and conjugate gradient computations. SIAM J. Matrix Anal. Appl. **13**(1), 121–137 (1992)

[23] Griewank, Andreas: The modification of Newton’s method for unconstrained optimization by bounding cubic terms. Technical Report NA/12, Department of Applied Mathematics and Theoretical Physics, University of Cambridge, (1981)

[24] Hestenes, Magnus R., Stiefel, Eduard: Methods of conjugate gradients for solving linear systems. J. Res. Natl. Bur. Stand. **49**(6), 409–436 (1952)

[25] Karimi, H., Nutini, J., Schmidt, M.: Linear convergence of gradient and proximal-gradient methods under the Polyak–Łojasiewicz condition. In: Joint European conference on machine learning and knowledge discovery in databases, pages 795–811. Springer, (2016)

[26] Lanczos, C.: An iteration method for the solution of the eigenvalue problem of linear differential and integral operators. J. Res. Nat. Bureau Standards, **45**(4), (1950)

[27] Liesen, Jörg., Strakoš, Zdenek: Krylov Subspace Methods: Principles and Analysis. Oxford University Press (2013)

[28] Liu, C., Zhu, L., Belkin, M.: Loss landscapes and optimization in over-parameterized non-linear systems and neural networks. Appl. Comput. Harmonic Anal. **59**, 85–116 (2022)

[29] Liu, Yang, Roosta, Fred: MINRES: from negative curvature detection to monotonicity properties. SIAM J. Optim. **32**(4), 2636–2661 (2022)

[30] Liu, Yang, Roosta, Fred: A Newton-MR algorithm with complexity guarantees for nonconvex smooth unconstrained optimization. arXiv preprint arXiv:2208.07095, (2022b)

[31] Łojasiewicz, Stanislaw: Une propriété topologique des sous-ensembles analytiques réels. Les équations aux dérivées partielles **117**, 87–89 (1963)

[32] Łojasiewicz, Stanislaw: Sur les trajectoires du gradient d’une fonction analytique. Seminari di geometria **115–117**, 1982 (1983)

[33] Luo, Zhi-Quan., Tseng, Paul: Error bounds and convergence analysis of feasible descent methods: a general approach. Ann. Oper. Res. **46**(1), 157–178 (1993)

[34] Meurant, G.: The Lanczos and conjugate gradient algorithms: from theory to finite precision computations. Society for Industrial and Applied Mathematics (2006)

[35] Meurant, Gérard., Strakoš, Zdeněk: The Lanczos and conjugate gradient algorithms in finite precision arithmetic. Acta Numer **15**, 471–542 (2006)

[36] Moré, Jorge J., Sorensen, Danny C.: Computing a trust region step. SIAM J. Sci. Stat. Comput. **4**(3), 553–572 (1983)

[37] Nesterov, Yurii, Polyak, Boris T.: Cubic regularization of Newton method and its global performance. Math. Program. **108**(1), 177–205 (2006)

[38] Nocedal, J., Wright, S.: Numerical Optimization. Springer, New York (2006)

[39] Paige, C C: The computation of eigenvalues and eigenvectors of very large sparse matrices. PhD thesis, University of London, (1971)

[40] Paige, Christopher C., Saunders, Michael A.: Solution of sparse indefinite systems of linear equations. SIAM J. Numer. Anal. **12**(4), 617–629 (1975)

[41] Parlett, B. N: The symmetric eigenvalue problem. Society for Industrial and Applied Mathematics (1998)

[42] Polyak, Boris T.: Gradient methods for the minimisation of functionals. USSR Comput. Math. Math. Phys. **3**(4), 864–878 (1963)

[43] Rebjock, Q., Boumal, N.: Fast convergence to non-isolated minima: four equivalent conditions for functions. arXiv preprint arXiv:2303.00096, (2023)

[44] Ring, Wolfgang, Wirth, Benedikt: Optimization methods on Riemannian manifolds and their application to shape space. SIAM J. Optim. **22**(2), 596–627 (2012)

[45] Steihaug, Trond: The conjugate gradient method and trust regions in large scale optimization. SIAM J. Numer. Anal. **20**(3), 626–637 (1983)

[46] Toint, P.: Towards an Efficient Sparsity Exploiting Newton Method for Minimization. In Sparse Matrices and their Uses, pp. 57–88. Academic press, (1981)

[47] Trefethen, L N, Bau, D.: Numerical linear algebra. Soc. Indus. Appl. Math (1997)

[48] Yuan, Yaxiang: On the truncated conjugate gradient method. Math. Program. **87**, 561–573 (2000)

[49] Yue, Man-Chung., Zhou, Zirui, Man-Cho So, Anthony: On the quadratic convergence of the cubic regularization method under a local error bound condition. SIAM J. Optim. **29**(1), 904–932 (2019)

[50] Zhou, Y., Wang, Z., Liang, Y.: Convergence of cubic regularization for nonconvex optimization under KŁ property. Adv. Neural Inform. Process. Syst., **31**, (2018)

